# The Day-to-Day Acute Effect of Wake Therapy in Patients with Major Depression Using the HAM-D_6_ as Primary Outcome Measure: Results from a Randomised Controlled Trial

**DOI:** 10.1371/journal.pone.0067264

**Published:** 2013-06-28

**Authors:** Klaus Martiny, Else Refsgaard, Vibeke Lund, Marianne Lunde, Lene Sørensen, Britta Thougaard, Lone Lindberg, Per Bech

**Affiliations:** 1 Psychiatric Centre Copenhagen, Rigshospitalet, Copenhagen University Hospital, Copenhagen, Denmark; 2 Psychiatric Research Unit, Mental Health Centre North Zealand, Copenhagen University Hospital, Copenhagen, Denmark; 3 Child and Adolescent Psychiatric Centre, Bispebjerg, Copenhagen University Hospital, Copenhagen, Denmark; Catholic University of Sacred Heart of Rome, Italy

## Abstract

**Background:**

This paper reports day-to-day data for from a one-week *intervention phase*, part of a 9-weeks randomised parallel study with patient having major depression (data from weekly visits have been reported). Wake therapy (sleep deprivation) has an established antidepressant effect with onset of action within hours. Deterioration on the following night’s sleep is, however, common, and we used daily light therapy and sleep time stabilisation as a preventive measure. In particular, we evaluated the day-to-day acute effect of and tolerance to sleep deprivation and examined predictors of response.

**Methods:**

Patients were assessed at psychiatric inpatient wards. In the wake group (n = 36), patients did three wake therapies in combination with light therapy each morning together with sleep time stabilisation. In the exercise group (n = 38), patients did daily exercise. Hamilton subscale scores were primary outcome (not blinded), secondary outcome was self-assessment data from the Preskorn scale and sleep.

**Results:**

Patients in the wake therapy group had an immediate, large, stable, and statistically significant better antidepressant effect than patients in the exercise group with response rates at *day5* of 75.0%/25.1% and remission rates of 58.6%/6.0%, respectively. The response and remission rates were diminished at *day8* with response rates of 41.9%/10.1% and remission rates of 19.4%/4.7%, respectively. Patients and ward personnel found the method applicable with few side effects. Positive diurnal variation (mood better in the evening) predicted a larger response to wake therapy. In the wake group napping on days after intervention predicted greater deterioration on *day8.*

**Conclusions:**

The intervention induced an acute antidepressant response without relapse between wake nights but with a diminishing effect after intervention. Development is still needed to secure maintenance of response. Avoiding napping in the days after wake therapy is important.

**Trial Registration:**

Clinical trials.gov NCT00149110

## Introduction

The objective of this study was to investigate if a chronotherapeutic intervention combining sleep deprivation (wake therapy) with light therapy and sleep time stabilisation could induce a rapid and augmented antidepressant response and remission in patients with major depression. The medium-term effect over a nine week period has been published elsewhere [1], whereby the wake therapy group manifested a sustained better response and remission than the exercise group. The present paper reports data from the one-week study period where patients were randomised to either wake therapy or exercise in an inpatient setting. In this study period patients had daily ratings of depression severity and did self-evaluation of sleep in order to reflect a timely assessment of any mood fluctuation and sleep changes. This was important as wake therapy is known to induce mood swings and alterations in sleep quality, length and phase.

Systematic clinical investigation has been performed on various aspects of the acute response to wake therapy for more than 40 years, and a large evidence base confirms the rapid antidepressant effect [2], [3], [4], [5]. The term wake therapy is used synonymous to sleep deprivation but has a more positive image for the patient. Deterioration or relapse after the first nights sleep after wake therapy (termed “recovery sleep”) has been and still is a problem, and several new methods have been tested, mainly in bipolar depressed patients. These include the use of wake therapy in combination with: bright light therapy [6], bright light therapy and sleep phase advance [7], bright light therapy and lithium [8], pindolol [9], bright light therapy and transcranial magnetic stimulation [10]. A manual [11] and an instruction chapter [12] assist clinicians in the practical details of chronotherapy with an emphasis on preventing relapse. The prevention of deterioration or relapse during a course of wake therapy is important with respect to its applicability, safety and acceptance. A fast response to an antidepressant intervention is very desirable but a steep relapse is equally undesirable. What we want from antidepressant therapy is a rapid and stable improvement.

The effect of wake therapy is linked to the phenomenon of diurnal variation [13], [14]. Slight alterations of sleep timing can cause dramatically changes in mood and at the extreme end of this is the positive response to an entire night of sleep deprivation. As the antidepressant improvement gained over night has been known to be unstable, the present protocol aimed at inducing day-to-day stability through the use of sleep time stabilisation and light therapy. Our earlier report of the results showed that this was attained as we found a smaller day-to-day variation of sleep parameters [1]. Napping is know from the literature to be depressiogenic after wake therapy [15] and was thus advised against.

This paper thus focuses on the acute effect of the interventions used in the one-week *intervention phase* as assessed throughout the intervention days on the inpatient ward, and on testing potential predictors of wake therapy response.

As the acute changes during or after wake therapy is very important for safety and tolerance we designed the *intervention phase* so as to be able to detect rapid changes in depression severity.

Thus the first objective regarding the *intervention phase* was:

- To investigate whether the combination of wake therapy, light therapy and sleep time stabilisation can prevent any deterioration *between* wake nights and/or *after* the end of the series of 3x wake therapy.
  As some discrepancy exists regarding the impact of diurnal variation on the effect of wake therapy response, patient self-assessed their mood over the course of the day for 6 days prior to the intervention week. Thus we were able to test our second objective:
- To investigate if the acute effect of wake therapy was influenced by the presence of diurnal variation of mood as assessed prior to the intervention?
  As the literature shows that napping after sleep deprivation is depressiogenic we sampled information regarding napping during the intervention days to test our third objective:
- To investigate to what extent daytime napping in the *intervention phase* caused deterioration of any achieved improvement?

## Methods

### General

The protocol for this trial and supporting CONSORT checklist are available as supporting information; see Checklist S1 and Protocol S1.

Methods have been described in detail in [1] which presented the medium-term results from weekly visits from a 9-weeks study period. Patients diagnosed with major depression were randomised in a ratio of 1∶1 into two parallel groups: the *wake* group or the *exercise* group. Inclusion criteria were: age above 18, major depressive episode, patients with bipolar disorder to be in mood stabilising treatment for at least one month in a recommended dosage and a HAM-D_17_ score ≥13. Exclusion criteria were: psychotic disorder, organic brain disorder, mental retardation, alcohol or drug abuse, pregnancy or insufficient contraception, light-induced migraine or epilepsy, retinal blindness or severe cataract, glaucoma, retinal diseases, antipsychotic drugs treatment, marked suicidal ideation, and severe agitation.

The study started with a one-week *run-in phase* where medication with duloxetine was begun and diurnal variation assessed.

Then followed a one-week *intervention phase* where all patients were admitted to an in-patient ward on a Monday (*day1)*. Patients randomised to the *wake* group carried out (A) three wake therapies alternating with recovery sleep nights, (B) daily morning bright light therapy and (C) sleep timing control. Patients randomised to the e*xercise* group were also admitted on a Monday (*day1*) and started a daily exercise program instructed by physiotherapists. Patients were all discharged on the following Saturday (*day6*) when patients in the *wake* group had carried out all 3 wake therapies. During each of the five days of the inpatient stay we assessed patients at the ward in the morning. After discharge patients were seen at the psychiatric research unit on the following Monday (*day8*).

This was followed by a 7-week *continuation phase* where patient in the *wake* group continued with sleep time stabilisation, daily morning light therapy and duloxetine 60 mg daily dosage and patients in the *exercise* group continued with an individual exercise program of at least 30 minutes duration and duloxetine in a daily dosage of 60 mg. In this paper we report data from the one-week *intervention phase.*

**Figure 1:**
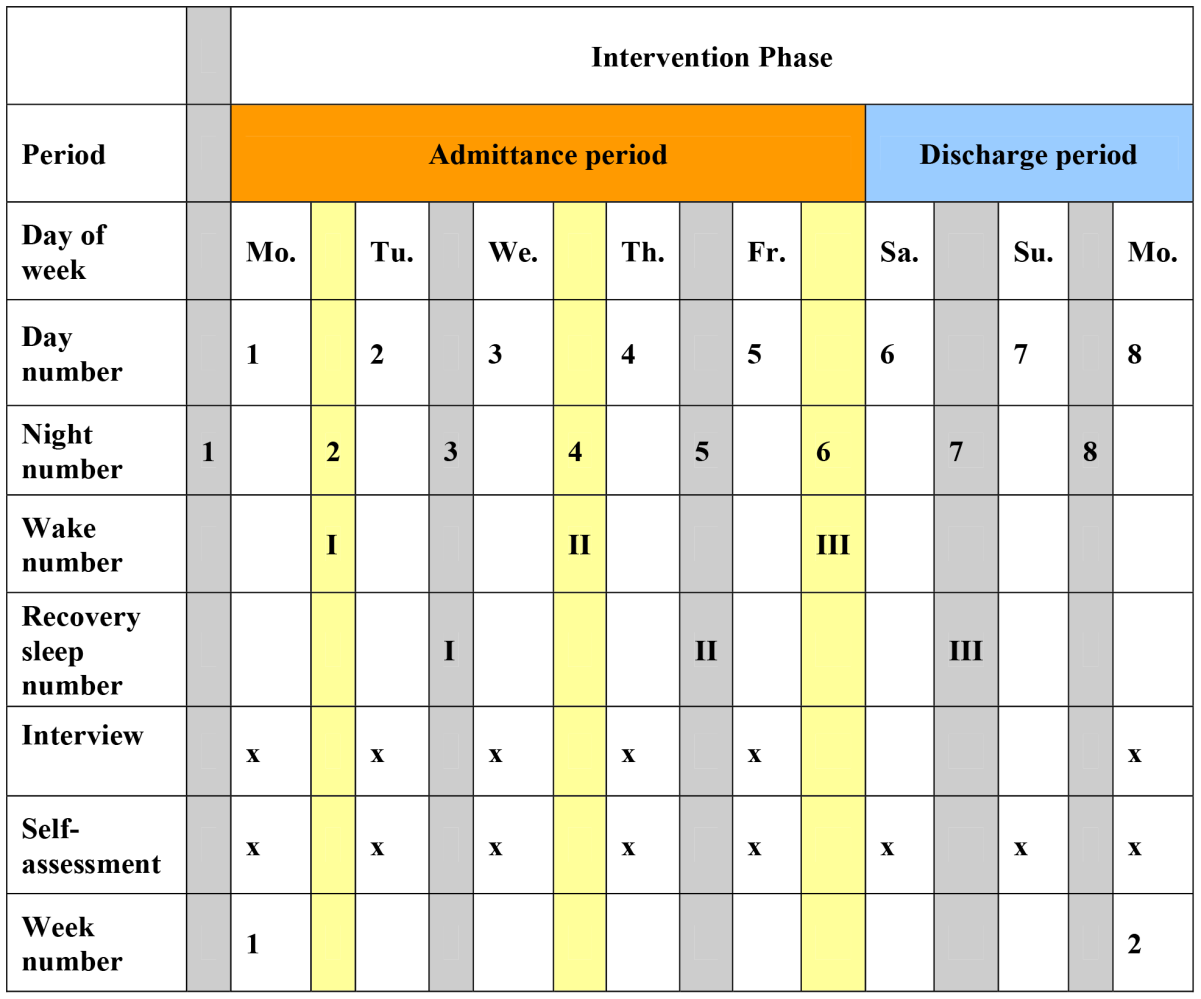
Consort diagram of subject flow.

The individual study days in the *intervention phase* and their relation to study procedures are named as shown in Figure 1: Monday is *day1,* Tuesday is *day2,* Wednesday is *day3,* Thursday is *day4,* Friday is *day5,* Saturday is *day6,* Sunday is *day7*, and the next day, Monday, is *day8*.

The intervention phase has, for the purpose of data analyses, been subdivided into a *admittance period* signifying the period from being admitted to the in-patients ward on a Monday (*day1*) and till being discharged on a Saturday (*day6*), and a *discharge period* signifying the period from discharge on a Saturday (*day6*) till assessments at the psychiatric research unit on the following Monday (*day8*).

### Wake Therapy

Wake therapies were scheduled for Mondays (*day1*), Wednesdays (*day3*) and Fridays (*day5*). Patients were instructed to stay up the entire wake nights and were encouraged not to sleep on the following day until 8 pm. Patients were allowed to walk freely in and outside the ward, take baths, talk to the staff, watch television, cook meals, listen to music, read, use the computer, and drink coffee etc. The ward staff was instructed to encourage patients to stay awake but without putting any pressure onto them. On Tuesday, Thursday and Saturday nights (recovery sleep I, II and III), patients were scheduled to go to sleep no later than 8 pm and to wake up no later than 8 am. This was used as a precaution, because oversleeping in the morning on recovery nights is known, from the literature, to worsen mood [16]. This adjustment to early sleep was intended to act like a milder version of a sleep phase advance, as this has been shown to facilitate antidepressant response [17].

### Light Therapy

Daily light therapy was administered daily for 30 minutes while on the in-patient ward; using a light box (SMIFA Biolamp, giving 10.000 lux white light at 40 cm distance from the screen). Individual timing was scheduled from an algorithm based on the patient’s score on the Morningness-Eveningness Questionaire (MEQ) [18] (but limited to 7 AM in the morning as the earliest). At 4 AM patients were administered 30 minutes of light to alleviate tiredness. Oral and written information on the light therapy procedure was given.

### Sleep Time Stabilisation

Guidance for sleep time stabilisation was based on daily entries in the sleep logs of sleep onset, sleep offset, subjective sleep quality (range 0 to 10 and 10 = best) and daytime naps and entries were used at daily and weekly visits to guide patients to keep a stable sleep-wake cycle. Patients who needed to take naps were instructed to schedule these at around 4 pm and not to exceed 30 minutes.

### Exercise

A daily exercise programme was started on admittance to the inpatient ward. Each participant planned a daily exercise program of minimum 30 minutes duration with the physiotherapist. Patients filled in daily logs with name of activity, perceived exertion [19] and duration of exercise. Patients in this group followed the ordinary bedtime and sleep length regime in the open ward. At the hospital, exercise was taken between 9 am and 4 pm.

### Medication

Patient received a fixed daily dosage of 60 mg duloxetine in the morning, begun at the run-in phase, a week prior to admittance to the in-patient ward.

### Structure of Reporting

**Figure 2:**
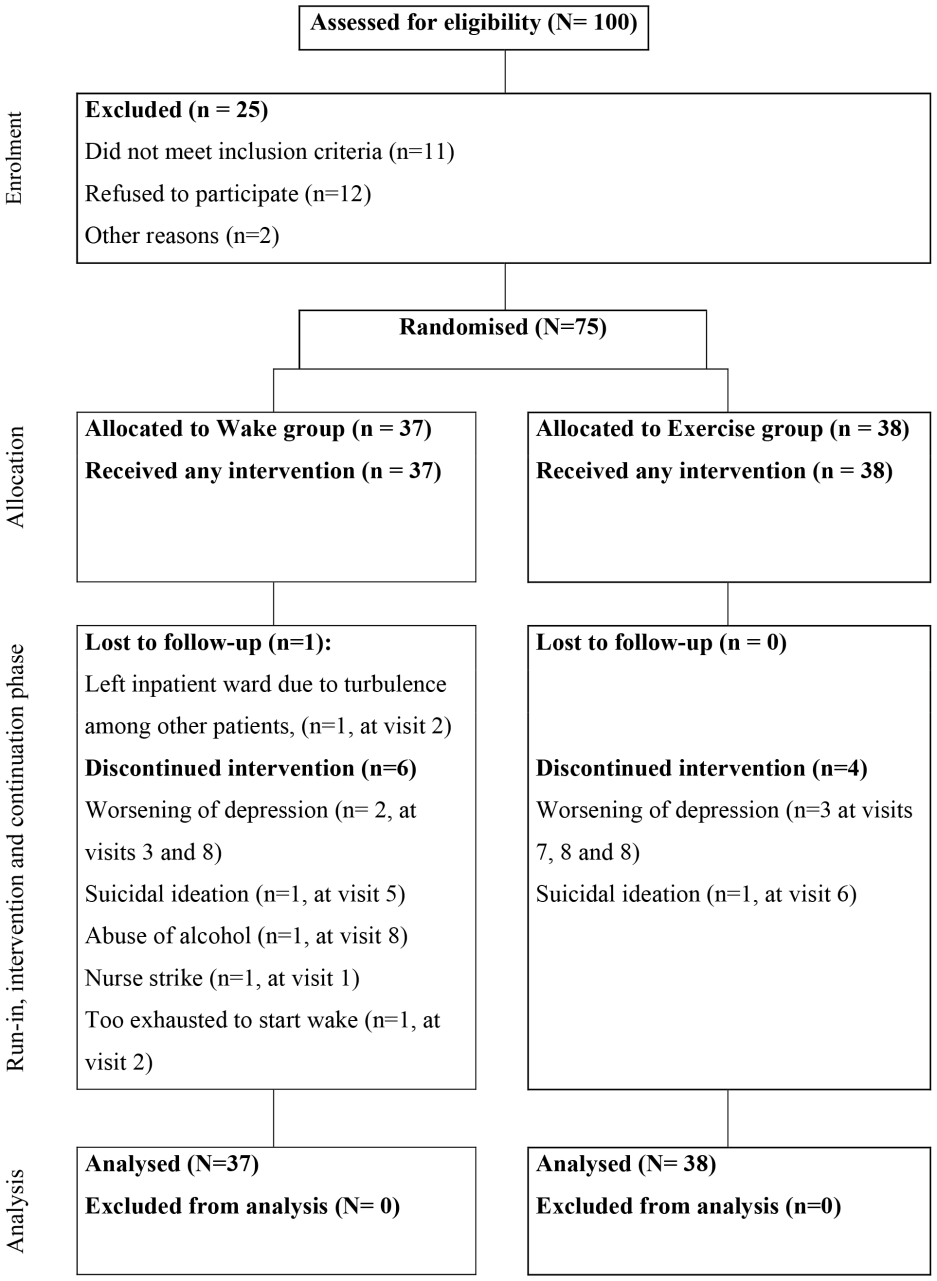
Flow chart and description of study procedures in the *intervention phase.*

Reporting followed the Consort guideline (Figure 2) [20].

### Approval, Monitoring and Ethics

Approvals were given by the committee on Biomedical Research Ethics, the Danish Medicines Agency and the Danish Central Data Register. Monitoring was done by the national GCP unit in Copenhagen. The study was carried out according to the declaration of Helsinki and the ICH-GCP guidelines [21].Written study information and oral description of the study were given to the patients before written informed consent was obtained.

### Recruitment

Patients were recruited from general practitioners and psychiatrists, open wards at the local mental health centre and a few patients through advertisements.

### Random Allocation Sequence

A randomisation list was made by an external statistician who by computer generated a random list with a block size of four (block size was blinded). A GCP qualified research coordinator labelled envelopes with consecutive numbers and inserted group specific instruction letters according to the randomization list (kept locked up). The envelope was handed over to the patient after signing the informed consent form.

### Blinding

Blinding of assessors at the daily assessments on the ward was not possible, as patients in the wake group would show signs of not having slept during wake nights. Assessments on the ward were thus done by other raters than assessments at weekly visits that were done by Hamilton raters blinded to treatment assignment.

### Sample Size

Sample size for the whole 9-week study was calculated from an expected reduction in HAM-D_17_ scores of 14 points from 24 to10 points (*wake group*) and of 11 points from 24 to 13 points (*exercise group*), and with a standard deviation of 6 points, a power of 80% to detect a significant difference (p = 0.05, two sided), in all 64 patients were needed in each group. This corresponds to an effect size of 0.50 (difference between groups/pooled standard deviation). As the expected difference between groups was hypothesized to emerge immediately when the wake and light therapy was given in the *intervention phase*, the sample size calculation from the main study also applied to this paper.

### Interim Analysis

No interim analyses were planned or performed.

### Stopping Rules

Criteria for discontinuation of study treatment was: a wish to discontinue treatment, intolerable or clinically significant side effects, a score of 15 or more on the Mania Scale (MAS), continued clinical worsening of depression, change of mood stabilizer in bipolar patients, and non-compliance with elements of the study.

### Study Registration

Study is registered at ClinicalTrials.gov with identifier NCT00149110.

### Outcomes

Outcome measures are described in detail in [1]. To assess depression severity we used the clinician-reported Hamilton depression rating scale 17 items version (HAM-D_17_). In the present analysis, focusing on the *intervention phase*, we have used the HAM-D_6_ [22], [23], [24], [25] subscale. This is a validated and unidimensional depression scale without any sleep items. Sleep items would be inaccessible in a wake therapy trial. The HAM-D_6_ contains six core items of depression from the HAM-D_17_, namely depressed mood, guilt feelings, work and interests, psychomotor retardation, psychic anxiety, and somatic general (tiredness and pain). The total score of the HAM-D_6_ can theoretically go from 0 (not depressed) to 22 (extremely depressed): a score of 12 equal severe depression, a score of 9 equals moderate depression. Remission on the HAM-D_6_ was defined as a score of 4 or less [26], response was defined as a reduction of 50% or more from baseline (*day1*) [27]. Deterioration rates were defined as scores equal to or above score values at *day1.* Deterioration was used as a measure of instability of depression severity. This HAM-D_6_ subscale does not include sleep items, making it appropriate for assessing depression severity over a course of wake therapies. The HAM-D_6_ interviews were performed at the ward in the morning on *day1* till *day5* and at the research unit on *day8*. The self-assessment scales (Preskorn and sleep logs) were administered on all days in the *intervention phase.* The Preskorn scale, constructed as a VAS scoring from 0 (no depression) to 10 (worst depression ever), was used to self-monitor mood changes during the day at the *run-in phase* and from day to day in the *intervention phase*. The Bech-Rafaelsen Mania scale (MAS) [22] was used to monitor any emergence of manic symptoms and was used on the same days as the HAM-D_6_.

Patients in the *wake* group filled in daily light therapy logs, daily sleep logs with sleep onset, sleep offset, and self-perceived quality of sleep [28], and the Stanford Sleepiness Scale [29], [30] for every hour of the wake nights.

Hospital staff filled in semi-qualitative evaluations of treatment elements during the *intervention phase* and patients did a similar evaluation at the following post intervention visit (*day8*).

During the *run-in phase*, patient self-assessed their mood on the Preskorn scale shortly after wake-up, then at 9 am, noon, 3 pm, 6 pm, 9 pm, and just before lights out - over a period of six days.

The rating window (time frame covered by the scale) [26] is for the HAM-D_6_ traditionally the past three days and this was used for assessment on *day1* and *day8*. On *day2* till *day5* the window for the HAM-D_6_ was limited to the past hour to improve sensitivity to changes in depression severity. The Preskorn scale was self-assessed at noon on all study days in the *intervention phase* (to minimize influence from diurnal variation).

Hamilton assessors were not blinded to patient’s treatment allocation. Assessors were certified for good inter-rater and test-retest reliability.

In the *intervention phase* we expected a rapid reduction in depression severity in the *wake* group and a more gradual reduction on the *exercise* group. The day-to-day variation in depression severity was unknown but thought to be of interest, as a large variation would be considered a substantial burden and hazard to patients.

The Morningness-Eveningness Questionaire (MEQ) was used to time light therapy according to individual patient chronotype [31]. In this paper we used it to examine any relation with depression outcome.

Patients were asked, at the beginning of the *run-in phase,* if in their present depressive episode retrospectively they had experienced a significant drop in mood after daytime naps, in order to investigate this as a predictor for effect of wake therapy.

### Data Analysis

All patients with data from any days of the *intervention phase* were included in analyses. Available data from HAM-D_6_ and Preskorn scales were computed within a Mixed Model Repeated Measures analysis [32]. This included for continuous endpoints (depression scores) a Random-effects Regression Model (RRM) and for dichotomous endpoint (response, remission, deterioration) a Generalized Estimation Equation model (GEE). Thus, for RRM, the model included depression score as output variable and baseline, day, treatment group, the interaction between day and treatment group as covariates. For GEE, the model included response, remission or deterioration fractions as output variables and baseline, day and treatment group as covariates. For tables and graphs estimated scores are presented. Baseline values (*day1*) are used to calculate estimated scores at subsequent days. Baseline values are mean scores from all patients, and are thus presented in tables as a single value for both groups. The model gives p-values for the whole of the *intervention phase* and post-hoc p-values for each day.

In this paper we have used *day1* of the *intervention phase* as baseline for analysis of days of the *intervention phase* (*day1 till 5*). The presented results from *day8* were calculated with *week0* (see Figure 1) as baseline with the model specified in [1] because *day8* is not part of the *intervention phase*.

To facilitate comparisons with other studies the unbiased effect size (Cohen’s) was calculated as described by Hegdes and Olkin [33]. For the Cohen effect size we used the method of last observation carried forward (LOCF) whereas in the RRM and GEE available data were used.

Analyses of diurnal variation of mood, based on Preskorn data from 7 times a day each day for 6 days self- assessed in the *run-in phase*, have been analysed in a similar mixed model with Preskorn scores as outcome variable and patient and interaction of patient with time as covariates. The resultant parameter estimate for time is used to represent the degree of diurnal variation and is in turn used in a RRM model as a covariate to analyse the influence of diurnal variation on depression scores. A positive diurnal variation of mood (with mood better in the evening) is defined as a time parameter estimate of less than zero (score reduction on the Preskorn scale = improvement) and a negative diurnal variation of mood (with mood worse in the evening) is defined as a parameter estimate of time greater than zero (score increase on the Preskorn scale = deterioration) both on the Preskorn scale. The MEQ sum score was entered as a covariate to examine its influence on depression outcome. Sleep parameters were analysed in a general linear model (GLM) with values at *day1* as baseline covariates except for the effect of sleep quality and napping that was analysed in a RRM model.

For the analyses of the impact of diurnal variation and naps on HAM-D_6_ scores at *day8* we included a separate covariate for *day8* as available data showed a non-linear deviation from other intervention days.

Primary outcome was remission rates at *day5* based on the HAM-D_6_ and outcomes for the other days are considered post-hoc. Secondary outcomes were response rates, deterioration rates and mean scores on the MAS, Preskorn and sleep parameters.

Analyses were performed by SAS 9.2 software. All time points are shown in the format of hour: minutes. Brackets after numbers are standard deviations unless otherwise stated. The level of statistical significance was set at a 5% level, two-sided.

## Results

### General

Patients were included from September 2005 to August 2008, last patient last visit March 2009. In all, 100 patients were screened and 75 patients were included in the study with 37 patients randomised to the *wake* group and 38 patients to the *exercise* group. Inclusion was stopped at 75 patients due to time constraints and funding limits. In the *wake* group one patient was kept waiting for the intervention, due to a nurse’s strike, and was in remission when the strike ended, and was thus discontinued and is not included in analysis. One patient in the *wake* group decided at visit two, before being admitted to the inpatient ward, not to go through with the wake therapies and was discontinued from the study but is included in analyses. One patient in the *wake* group did not fill in sleep logs and left the inpatient ward after one wake therapy due to perceived uneasiness on the ward but is included in the analyses. Thus 36 of the patients allocated to the wake group and all 38 patients allocated to the exercise group were included in the data analyses. In the wake therapy group only 34 patients had available sleep data. Due to logistic reasons the number of patients assessed with the HAM-D_6_ in the exercise group was fewer on days 4 and 5. Numbers are given in tables. No patients discontinued in the *exercise* group. The number of performed sleep deprivations in the *intervention phase* was 35 for wake I, 34 for wake II and 28 for wake III. Thus overall 97 wake therapy nights were carried out in the *intervention phase*. Patient performed exercise with a mean duration of 51.2 (SD = 45.0) minutes/day. The mean Borg scale score was 13.1 (SD = 6.1) corresponding to moderate exertion.

### Deterioration and Depression Outcome

The fraction of patients having a deterioration defined as a HAM-D_6_ scale score above the entry level of 12 points, at any of the assessed days, was very low and below 4.2% in the *exercise* group and 1.8% in the *wake* group. The difference in deterioration between groups was statistically non-significant for the whole period.

Table 1 shows estimated post *day1* remission and response rates for assessed intervention days. Clinically relevant and statistically significant larger response and remission rates were seen in the *wake* compared to the *exercise* group from *day2* and reaching a maximum at *day5* with response rates in the wake/exercise groups of 75.0%/25.1% and remission rates of 56.8%/6.0%. The difference between groups was statistically significant (response: Odds ratio 9.0; CL 3.7–21.8, p<.0001 and, remission: Odds ratio 20.8; CL 5.6–77.1, p<.0001) and post hoc for each assessed day (see Table 1).

**Table 1:** Estimated Post-*Day1* mean Response and Remission rates based on HAM-D_6_ scores for Each Treatment Group by day.

	Response Per cent (n)				
	Wake %	Exercise %	Odds Ratio	95% CL	P Value
	(n)	(n)			
*Day1*(baseline)	0 (0/36)	0 (0/38)	1	-	-
*Day2* (after wake I)	58.7 (16/31)	13.7 (8/35)			
*Day3* (after recovery sleep I)	64.6 (23/35)	16.9 (3/34)	9.0*	3.7–21.8	<.0001
*Day4* (after wake II)	70.1 (21/31)	20.7 (6/26)			
*Day5* (after recovery sleep II)	75.0 (24/30)	25.1 (4/22)			
	**Remission Per cent (n)**				
	**Wake %**	**Exercise %**	**Odds Ratio**	**95% CL**	**P Value**
	**(n)**	**(n)**			
*Day1* (baseline)	0 (0/36)	0 (0/38)	1	-	-
*Day2* (after wake I)	38.6 (11/31)	2.9 (1/35)			
*Day3* (after recovery sleep I)	44.6 (18/35)	3.7 (2/34)	20.8*	5.6–77.1	<.0001
*Day4* (after wake II)	50.7 (15/31)	4.7 (2/26)			
*Day5* (after recovery sleep II)	56.8 (17/30)	6.0 (0/22)			

Numbers of patients with response and remission given in parenthesis.

Abbreviations: HAM-D_6_ = Hamilton Depression Rating Scale subscale, Response as a reduction of more than 50% from *day1,* Remission was defined as a HAM-D_6_ score below 5, CL = confidence limits.

* OR was from the regression model without interaction between day and intervention and the main effect of intervention was reported.

The response rates at *day8* ( = week2), as analysed on the 9-weeks dataset was reduced to 41.9%/10.1% in the wake/exercise groups and remission rates were reduced to 19.4%/4.7%.The difference between groups at *day8* was statistically significant response: Odds ratio 6.4; CL 2.3–17.4, p = .0002 and, remission: Odds ratio 4.9; CL 1.4–17.0, p = .01).

Table 2 shows estimated post d*ay1* HAM-D_6_ scores for assessed intervention days. A significant and clinically larger reduction in scores was seen in the *wake* group compared to the *exercise* group from *day2* (the day after the first wake therapy night) with a score difference of 2.5 (SE 0.7), (95% CL, 1.1–3.9, p = .0007) and the score differences increased on the following days to a maximum of 4.6 (SE 0.6) on *day5* (95% CL, 3.4–5.9,p<.0001) solely due to a further reduction in scores in the *wake* group, whereas the scores in the *exercise* group were unchanged. The scores at *day5* were 4.1 (SE 0.4) in the *wake* group and 8.7 (SE 0.5) in the *exercise* group. The difference in HAM-D_6_ scores between groups was significant for the interaction between groups and days (F_181_ = 8.8, p = .004) and post hoc for each assessed day. The post hoc scores, estimated from the nine weeks dataset, at *day8* were 7.5 (SE 0.5) in the wake group and 9.7 (SE 0.4) in the exercise group (F_529_ = 3.4, p<0.0007), (see Table S2). The difference between groups had an effect size (Cohen’s) of 1.43 (CL 0.92–1.94) at *day5* and 0.53 (CL 0.06–0.99) at *day8*.

**Table 2:** Estimated Mean Post-*Day1* HAM-D_6_ scores for Each Treatment Group by day. Numbers of patients given in parenthesis.

	Wake (SE)	Exercise (SE)	Difference Between Groups		
	[n]	[n]			
Day	Mean (SE)	Mean (SE)	Score (SE)	CL	P value
Day1	12.1 (0.2)		-		NA
	[Wake 37 Exercise 38]				
Day2 (after wake I)	6.2 (0.5)	8.7 (0.5)	2.5 (0.7)	1.1–3.9	.0007
	[31]	[35]			
Day3 (after recovery sleep I)	5.5 (0.4)	8.7 (0.4)	3.2 (0.6)	2.0–4.4	<.0001
	[35]	[3]			
Day4 (after wake II)	4.8 (0.4)	8.7 (0.4)	3.9 (0.5)	2.8–5.0	<.0001
	[31]	[26]			
Day5 (after recovery sleep II)	4.1 (0.4)	8.7 (0.5)	4.6 (0.6)	3.4–5.9	<.0001
	[30]	[22]			

Abbreviations: HAM-D6 = Hamilton Depression Rating Scale, NA = not applicable, SE = standard error, CL = confidence limits.

Table 3 shows estimated baseline-adjusted Preskorn scale scores. A better outcome was seen in the wake group compared to the exercise group from *day1* through till *day8* and in contrast to results from the HAM-D_6_ scale, no tapering of the difference between groups or deterioration from *day5* till *day8* was seen. The difference between scores in the groups was significant for the whole period (F_69_ = 11.5, p = .001) and post hoc for all days.

**Table 3:** Estimated Mean Post-D*ay1* Preskorn scores for Each Treatment Group by day.

	Wake	Exercise	Difference Between Groups		
Day	Mean (SE)	Mean (SE)	Score (SE)	CL	P value
Day1	5.6 (0.3)[Wake 34 Exercise 38]		-		NA
Day2 (after wake I)	4.1 (0.2) [34]	5.2 (0.2) [38]			
Day3 (after recovery sleep I)	4.1 (0.2) [34]	5.1 (0.2) [38]			
Day4 (after wake II)	4.0 (0.2) [34]	5.0 (0.2) [38]			
Day5 (after recovery sleep II)	3.9 (0.2) [34]	4.9 (0.2) [37]	1.0 (0.3)*	0.4–1.7	0.001
Day6 (after wake III)	3.8 (0.2) [34]	4.9 (0.2) [38]			
Day7 (after recovery sleep III)	3.7 (0.3) [34]	4.8 (0.3) [37]			
Day 8 (week 2)	3.7 (0.3) [32]	4.7 (0.3) [37]			

Numbers of patients given in square brackets.

Abbreviations: NA = not applicable, SE = standard error, CL = confidence limits.

* Score difference was from the regression model without interaction between day and intervention and the main effect of intervention was reported.

MAS scale scores (excluding item five, sleep) showed that no patient reached any level of mania. The maximum score was below 5 (below cut-off for mild mania).

Significantly more of those patient that had obtained response at day two were also in response at day eight compared to patients non-responding at day two (Fisher’s Exact test; p = 0.05). The positive predictive value (the probability that patients responding after the first wake therapy maintained the response at day eight) was 56.3% and the negative predictive value (the probability that patients not responding after the first wake therapy did also not respond at day eight) was 75.0%.

### Diurnal Variation, Mood and Chronotype

Data on diurnal variation of mood, as assessed by the Preskorn scale, was present for 72 patients from the run-in week (reduced scores indicates improvement in the Preskorn scale). A statistically significant variation of mood was found for time of day (F_344_ = 4.9, p<.0001) with no significant differences between treatment groups. The diurnal variation (mean of 6 days) ranged from a maximum score increase of 3.8 points (worsening during the day) to a maximum score reduction of 3.6 (improvement during the day). When dividing into positive *or* negative diurnal variation we found that positive diurnal variation (morning worst) was present in 58.3% of patient and negative diurnal variation (evening worst) scores was present in 41.7% of patients.

We analysed whether diurnal variation of mood at the run-in phase was predictive of the HAM-D_6_ depression scores during the intervention phase and found that the degree of diurnal variation had a significantly *differential* impact on scores in the two groups (F_63_ = 14.2, p = 0.0004 for the interaction).

We then examined the magnitude of impact of diurnal variation of mood separately for the two groups, by using the 1. Quartile (positive diurnal variation), the median, and the 3. Quartile (negative diurnal variation) values of the diurnal parameter estimate range (Q_1_ = –0.1976, median = 0.0363, and Q_3_ = 0.1001) as covariates in the model with *day1* as baseline. At *day5* patients in the *wake* group with positive diurnal variation (Q_1_) had an estimated HAM-D_6_ score of 3.5 (SE 0.5), patients with a small diurnal variation (median) had a score of 4.2 (SE 0.4), and patients with a negative diurnal variation of mood (Q3) had a score of 4.7 (SE 0.5).

Correspondingly at *day5* patients in the exercise group with positive diurnal variation (Q_1_) had a mean score of 9.6 (SE 0.6), patients with a small diurnal variation (median) has a score of 8.9 (0.5) and patients with a negative diurnal variation (Q3) a score of 8.4 (SE 0.6). Positive diurnal variation thus improved outcome in the wake group but worsened outcome in the exercise group and negative diurnal variation worsened outcome in the wake group and improved outcome in the exercise group, compared to patients without any clinical diurnal variation of mood (median).

The total scores from the Morningness-Eveningness Questionnaire (MEQ) was analysed as a covariate in the RRM model. Analysis showed no significant impact on depression scores.

### Sleep and Influence of Naps on Depression Outcome

At the start of study patients were asked to rate retrospective whether they, in their current depressive episode, had experienced any mood drop after having a daytime nap. Results from this assessment showed that a “nap-mood-drop” had been present in 47.1% of patient and had lasted a mean of 92.7 (69.5) minutes after end of nap; equally prevalent in both groups. The presence of a mood drop after daytime napping had a significantly negative influence on HAM-D_6_ scores (F_65_ = 3.9, p = 0.05) in the intervention phase with no difference between groups.

Results from the sleep logs are given in Table S3. This shows that, during the *intervention phase*, sleep onset was advanced in both treatment groups but significantly more in the wake group with 79.6 (99.4) minutes compared to 39.7 (125.6) minutes in the exercise group (between groups F_1_ = 10.1, p = .002). Sleep offset was advanced in the wake group by 30.0 (89.1) minutes but not in the exercise group where a slight sleep delay of 3.0 (132.8) minutes was found (between groups F_1_ = 0.6, p = 0.43). Sleep duration was increased in both groups: 49.6 (126.9) minutes in the wake group and 42.7 (123.0) minutes in the exercise group (between groups: F_1_ = 2.6, p = 0.11). The mean sleep duration for patients in the *wake* group on nights after wake therapy (recovery sleep nights I, II, III: *night3*, *night5* and *night7*), was: *night3* with 10∶35 (1∶11) hour: minutes, *night5* with 10∶14 (1∶41) hour: minutes and *night7* with 9∶51 (1∶58) hour: minutes. Sleep duration on these nights were significantly longer than corresponding sleep days in the exercise group that were: *night3* with 7∶17 (1∶20) hour: minutes (between groups: F_1_ = 119.2, p<.0001), *night5* with 7∶47 (1∶35) hour: minutes (between groups: F_1_ = 36.0, p<.0001), and *night7* with 7∶17 (1∶48) hour: minutes (between groups: F_1_ = 29.4, p<.0001). Scores on self-perceived quality of sleep increased in both groups but significantly more in the wake group (F_1_ = 10.5, p = 0.002). The total mean sleep length for the eight nights (including sleep for those not taking the third wake therapy) was 49∶45 (6∶03) hour: minutes in the wake group and 60∶02 (7∶45) hour: minutes in the exercise group (excluding naps in both groups).

Any change of sleep-offset from end of the *admittance period* to the end of the *discharge period* did not have any significant influence on HAM-D_6_ scores at *day8* (F_71_ = 0.08, p = 0.77).

Sleep logs showed that 41.2% (n = 14/34, with a total of 25 naps) of patients in the *wake* group and 52.6% (20/38, with a total of 60 naps) of patients in the *exercise* group napped in the *intervention phase*. The mean duration of the 25 naps in the *wake* group was 96.0 (65.5) minutes and the mean duration of the 60 naps in the *exercise* group was 75.3 (45.4) minutes. In the *wake* group, the total amount of napping time, during the *intervention phase*, was 2400 minutes distributed with 675 minutes (n = 9) in the *admittance period* (*day1* till *day5*) and 1725 minutes (n = 9) in the *discharge period* (*day6* till *day8*). In the exercise group, the total amount of napping time was 4520 minutes distributed with 2380 minutes (n = 16) in the *admittance period* (*day1* till *day5*) and 2140 minutes (n = 15) in the *discharge period* (*day6* till *day8*). The mean sleep midpoint of naps was 14∶31 (2∶55) in the *wake* group and 13∶52 (2∶50) in the *exercise* group. Only 14.7% (n = 5) of patients in the *wake* group were napping on the days after a wake night; one patient after the first wake, three patients after the second wake night and one patient after the third wake night. All differences between groups on nap statistics were non-significant.

We then examined the effect of napping on depression outcome (HAM-D_6_ scores) in the *intervention phase* and found that napping significantly worsened depression scores (F_70_ = 4.4, p = 0.04) in the whole group. Further analysis showed that this worsening was predominantly due to napping in the *discharge period* where napping significantly worsened depression outcome (F_70_ = 9.2, p = 0.003) where as napping in the *admittance period* had a small and nonsignificant worsening effect on depression scores. (F_70_ = 1.1, p = 0.3). Furthermore the worsening of depression scores seen in the *discharge period* was predominantly in the wake group (F_69_ = 5.8, p = 0.02).

**Figure 3:**
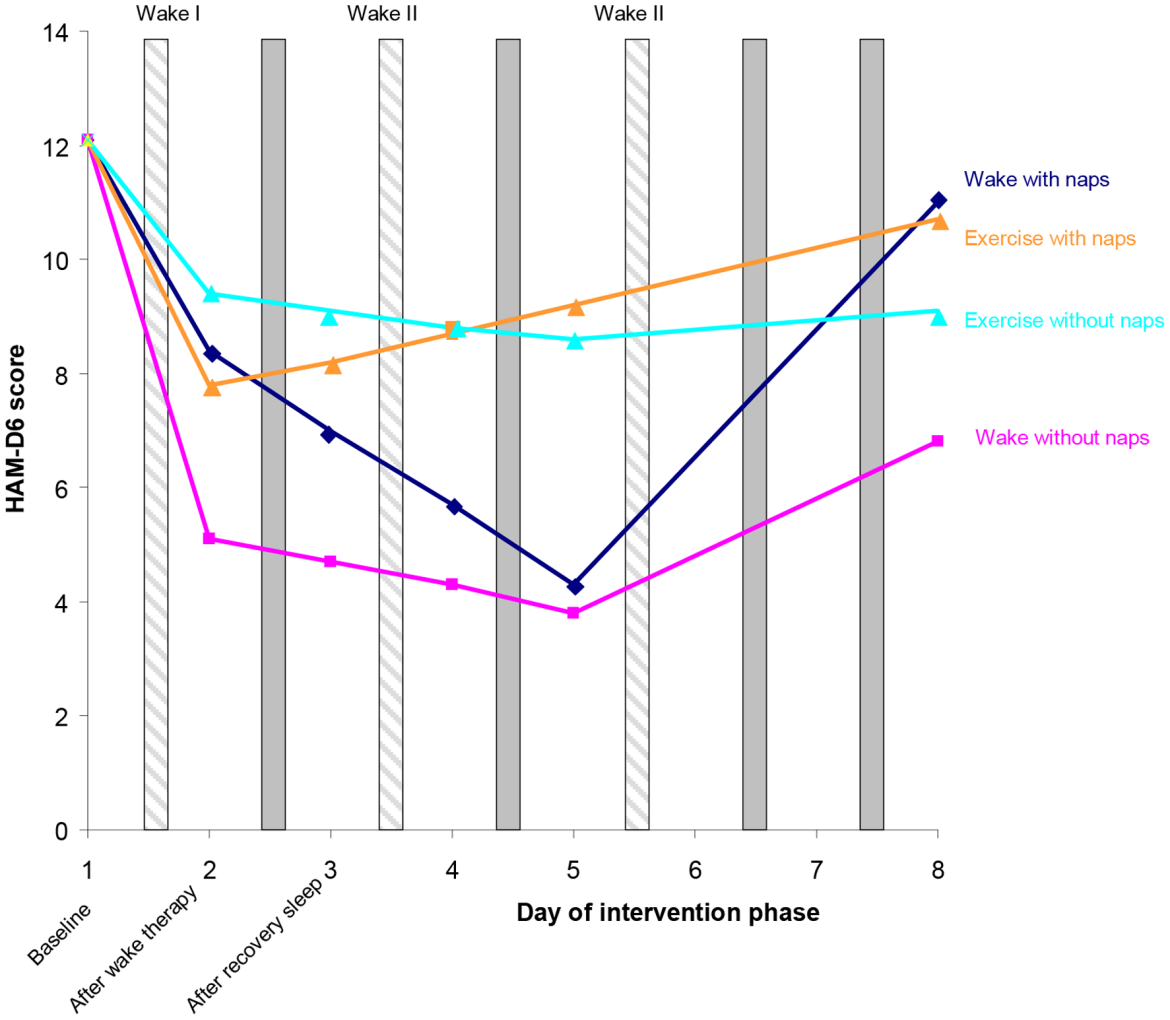
Estimated Mean Post-*day1* HAM-D_6_ scores by napping status in the discharge period (*day6* till *day8*) by treatment group.

To illustrate the effect of napping in the *discharge period* on depression scores in the two treatment groups we calculated the baseline adjusted (*day1*) estimated HAM-D_6_ scores for all days in the *intervention phase* for patients who napped (n = 24) and for patients who did not nap in this period. Results are shown in Figure 3. The main finding is that patients in the *wake* group who napped in the *discharge period* had a very large deterioration from *day5* till *day8* whereas patients not napping in the *wake* group had a much smaller deterioration in the same period. Also, in the wake group, we found that patients napping in the *discharge period* had a moderately *lesser* effect of wake therapy during the *admittance period* (F_65_ = 3.8, p = 0.06) compared to those patients without napping. Difference in depression scores in the exercise group between those napping and those not napping were nonsignificant.

### Patient and Staff Evaluations

Table 4 shows patients and staffs evaluation of study procedures. In general there was a high level of global satisfaction with the study procedures. Additional semi-qualitative data (not shown in Table 4) showed that conversations with investigators were rated especially beneficial in 37.9% of wake patients and 46.0% of exercise patients. In the wake group 20.7% found wake therapy especially beneficial and 48.0% found it difficult. In the exercise group 35.1% found exercise especially beneficial and 24.3% found it difficult.

**Table 4:** Patient and staff evaluations of procedures (wake, sleep time stabilisation, light and exercise) used in intervention week.

	Wake	Exercise
Patients’ evaluation	Per cent (n)	Per cent (n)
Felt global improvement	87.9 (29/33)	81.1 (30/37)
Satisfied with study procedures	81.8 (27/33)	94.6 (35/37)
Did you find any study procedure especially beneficially	87.9 (29/33)	100 (37/37)
Did you find any study procedure especially disagreeable	72.7 (24/33)	70.3 (26/37)
**Day-time staff evaluation**		
Staff evaluated that patient improved during stay at ward	82.6 (19/23)	52.6 (10/19)
Staff evaluated that some of the used study procedures were difficult for patient	73.9 (17/23)	52.6 (10/19)
Staff evaluated that some of the used study procedures were beneficial for patient	86.4 (19/22)	89.5 (17/19)
Staff indicated that study procedures are applicable in ward	95.9 (21/22)	57.9 (11/19)
Staff indication that study procedures could be used in ward as a treatment option for patients with depression	81.8 (18/22)	94.7 (18/19)
**Night-watch staff evaluation**		
Staff evaluated that patient improved during stay at ward	47.4 (9/10)	NA
Staff evaluated that used study procedures were difficult for patient	52.4 (11/21)	NA
Staff evaluated that used study procedures were beneficial for patient	75.0 (15/20)	NA
Staff indicated that study procedures are applicable in ward	70.0 (14/20)	NA
Staff indication that study procedures could be used in ward as a treatment option for patients with depression	85.0 (17/20)	NA

Per cent refers to positive responses.

The following supplemental data provide further information about the study:

Supplemental tables and figures:

Table S1: sociodemographics showing data for antidepressants, depression history, and age and length of depression in past 5 years. No significant differences were found between groups.

Table S2: baseline-adjusted estimated mean HAM-D_6_ scores by treatment group from the medium-term 9-weeks study for comparison. The magnitude of difference between groups remained stable for the whole period. The difference in HAM-D_6_ scores between groups was significant (F_529_ = 8.9, p = .003) and also post hoc for all weeks. The interaction between group and week was insignificant indicating a parallel reduction in scores in the two groups;

Figure S1: time course of depression ratings on the HAM-D_6_ scale for individual patients and days in the *interventions phase* by treatment group;

Figure S2: sleepiness as measured on all wake nights by the Stanford Sleepiness Scale (mean± standard deviation), LOCF. Mean sleepiness at 11 PM was 2.3 (1.1) (corresponding to: “functioning at high levels, but not at peak; able to concentrate”) and increased by each hour to a maximum of 4.0 (1.9) (corresponding to: “somewhat foggy, let down”) at 6 PM and then gradually diminished to 3.3 (1.6) (corresponding to: “awake, but relaxed; responsive but not fully alert”) at 8 PM.

## Discussion

The presented results confirm our *first* hypothesis: that a combination of wake therapy, light therapy and sleep time stabilisation can induce a rapid and marked response and remission without any relapse or deterioration between intervention days. It is thus a replication of the results found by other authors [34].

By necessity, the rating period during the intervention days is short and scores cannot be directly compared with scores from weekly assessments (*day1* and *day8*) where the standard retrospective rating period of three days has been used. However, there is no doubt that the marked response and remission rates seen immediately after the first wake therapy and augmented on the following intervention days are diminished somewhat on the following weekly assessment (*day8*). Thus the full goal of the study is not accomplished. In this respect it is important to notice that the dreaded mood fluctuations between wake nights and after recovery sleep, seen in some earlier studies using wake therapy, were not seen in this study.

The regime was well tolerated but as mentioned in the previous report [1] a few patients experienced anxiety attacks following wake therapy nights. Sleep loss due to wake therapies when compared to sleep duration in the *exercise* group was moderate and confirms that wake therapy does not cause a huge sleep deficit but is more a reallocation of sleep.

Concerning our second hypothesis, we found, as expected, that the presence of a positive diurnal variation (better in the evening) was associated with a better treatment response in the wake group. It is not established whether the diurnal variation of mood can be regarded as a continuum, as we implicitly have done, of a single latent biological parameter or whether there is a more complex and individual biology at the core of the phenomenon.

Gordijn et al found that variability in itself more than any definite type of variation predicted response to sleep deprivation [35]. In our opinion a positive diurnal variation should though still be recommended as a positive predictor when considering a patient for wake therapy regimens.

The effect size on *day5* of 1.45 is considered very large and larger than seen in drug therapy trials and comes within 5 days.

Regarding our third hypothesis we could confirm [15] that napping, after the wake therapy regimen, is associated with a much greater tendency for deterioration than for patients not napping. Thus, when using this wake paradigm, advice should be given and control measures employed to avoid any napping in the days following wake therapy. We cannot from this study with certainty conclude that napping by itself was the cause of the greater deterioration as inherent patient characteristics could play a role. The finding that patients who did nap after the end of the wake therapy regimen also had less effect during the wake therapies, suggests that the tendency to nap may be linked to some degree to the non-response to wake therapy.

The sleep logs showed a significant advance of the sleep-wake cycle in the wake group, which might also have contributed to the antidepressant effect.

Performing a series of three wake therapies is quite demanding on the patients and even though there is a substantial gain in the short and a moderate gain after nine weeks it would still be very useful to be able to predict which patients will benefit the most from this intervention. Our analysis showing a high negative predictive value suggests that non-response to an initial wake therapy gives little hope of response after further wake therapies. This finding could be useful guiding the clinicians.

It is a limitation of the study that the statistical analysis had to be performed within a larger dataset and with the use of another baseline than for the 9-weeks study. As the scores at week 1 (*day1*) were quite similar across groups we do not suspect this to be a major problem. Fewer patients were assessed in the exercise group with the HAM-D_6_ at days 4 and 5 primarily due to logistic reasons pertaining to exercise training sessions. As this was not related to depression severity we do not believe this has biased the results.

Due to the rather small sample size statistical significance of sub-analyses might be due to change findings of multiple testing and should thus be interpreted with caution.

Depression ratings from the days of the intervention week were based on a ‘last hour’ time window and this might bias towards lower scores compared to scores from longer time windows. This would give an artificially deterioration at end of intervention week where rating were based on a last ‘three days time’ window.

Some imprecision might come from performing ‘last hour’ rating on slightly different time point of the day.

As patients in this study were predominantly treatment resistant [1] the findings cannot be generalised to non-treatment resistant patient and as the majority of patient were unipolar we cannot generalise findings to bipolar patients.

## Supporting Information

Figure S1 **Individual patient’s available data from the HAM-D6 scale for the 8 days of the interventions phase for each patient by treatment group.**
(DOC)

Figure S2 **Sleepiness as measured on wake nights by the Stanford Sleepiness Scale (mean± standard deviation) for 94 wake nights (data missing from 3 nights), LOCF.** Scoring: 1 = Feeling active, vital, alert, or wide awake, 2 = Functioning at high levels, but not at peak; able to concentrate, 3 = Awake, but relaxed; responsive but not fully alert, 4 = Somewhat foggy, let down, 5 = Foggy; losing interest in remaining awake; slowed down, 6 = Sleepy, woozy, fighting sleep; prefer to lie down, 7 = No longer fighting sleep, sleep onset soon; having dream-like thoughts.
(DOC)

Table S1 **Sociodemographics.**
(DOC)

Table S2 **Baseline-adjusted estimated mean HAM-D6 scores by treatment group from the medium-term 9-weeks study.**
(DOC)

Table S3 **Results from the sleep logs.**
(DOC)

Checklist S1 **Consort checklist.**
(DOC)

Protocol S1 **Study protocol.**
(DOC)
