# Correction: The Day-to-Day Acute Effect of Wake Therapy in Patients with Major Depression Using the HAM-D_6_ as Primary Outcome Measure: Results from a Randomised Controlled Trial

**DOI:** 10.1371/annotation/3dcdb46a-e2b7-4305-8025-ce267688e9d6

**Published:** 2013-10-16

**Authors:** Klaus Martiny, Else Refsgaard, Vibeke Lund, Marianne Lunde, Lene Sørensen, Britta Thougaard, Lone Lindberg, Per Bech

The titles and legends for Figures 1 and 2 were incorrectly switched. The title and legend currently appearing with Figure 1 belong with Figure 2, and the title and legend currently appearing with Figure 2 belong with Figure 1. The figures themselves are in correct order. 

