# Supplementary material for: The Day-to-Day Acute Effect of Wake Therapy in Patients with Major Depression Using the HAM-D6 as Primary Outcome Measure: Results from a Randomised Controlled Trial
Source: PLoS One. 2013 Jun 28;8(6):e67264. doi: 10.1371/journal.pone.0067264 (PMC3696105; doi:10.1371/journal.pone.0067264)
Supplement: Table S1 — Sociodemographics. (DOC) [file pone.0067264.s003.doc]

# Table S1. Baseline Clinical and Demographic Characteristics from start of study

|  | Treatment Group | | | | | |
| --- | --- | --- | --- | --- | --- | --- |
|  | Wake N=37 | | | Exercise N=38 | | |
|  | N | % |  | N | % |  |
| Female | 24 | 64.9 |  | 20 | 52.6 |  |
| Treatment with antidepressants at inclusion | 31 | 83.8 |  | 32 | 84.2 |  |
| Previously treated with antidepressants for MDE | 21 | 56.8 |  | 21 | 55.3 |  |
|  | Mean | SD | Range | Mean | SD | Range |
| Age (years) | 46.9 | 12.6 | 21-70 | 48.5 | 11.2 | 23-69 |
| Age at first MDE (years) | 32.7 | 15.9 | 10-66 | 32.6 | 14.0 | 9-68 |
| Number of past MDE’s | 8.7 | 11.0 | 0-40 | 6.2 | 7.5 | 0-30 |
| Duration of current MDE (months) | 24.6 | 29.0 | 1-120 | 21.3 | 54.3 | 1-288 |
| Time spend depressed past five years (months) | 32.8 | 20.3 | 3-60 | 24.6 | 20.3 | 1-60 |
| Time spend manic past five years (months) | 1.2 | 5.1 | 0-27 | 0.5 | 1.6 | 0-7 |
| Time spend euthymic past five years (months) | 25.9 | 20.7 | 0-57 | 34.7 | 20.0 | 0-59 |
