# Supplementary material for: The Day-to-Day Acute Effect of Wake Therapy in Patients with Major Depression Using the HAM-D6 as Primary Outcome Measure: Results from a Randomised Controlled Trial
Source: PLoS One. 2013 Jun 28;8(6):e67264. doi: 10.1371/journal.pone.0067264 (PMC3696105; doi:10.1371/journal.pone.0067264)
Supplement: Table S2 — Baseline-adjusted estimated mean HAM-D6 scores by treatment group from the medium-term 9-weeks study. (DOC) [file pone.0067264.s004.doc]

**Table S2. Estimated Mean Post-baseline HAM-D_6_ scores for Each Treatment Group by week (Intervention phase between week 1 and 2). Numbers of patients given in parenthesis.**

|  | **Wake [n]** | **Exercise [n]** | **Difference Between Groups** | | |
| --- | --- | --- | --- | --- | --- |
| **Week** | **Mean (SE)** | **Mean (SE)** | **Score (SE)** | **CL** | **P-value** |
| Week0* | 12.9 (0.2)  [Wake 37 Exercise 38] | | - | | NA |
| Week1 | 11.6 (0.5)  [36] | 12.1 (0.5)  [38] | 0.4 (0.7) | 1.7-(-0.9) | 0.55 |
| Week2 | 7.5 (0.5)  [34] | 9.7 (0.4)  [38] | 2.2 (0.6) | 3.5-0.9 | 0.0007 |
| Week3 | 7.2 (0.4)  [32] | 9.4 (0.4)  [38] | 2.2 (0.6) | 3.3-1.1 | <.0001 |
| Week4 | 6.8 (0.3)  [33] | 9.0 (0.3)  [37] | 2.2 (0.5) | 3.1-1.2 | <.0001 |
| Week5 | 6.5 (0.3)  [32] | 8.7 (0.3)  [34] | 2.2 (0.4) | 3.0-1.3 | <.0001 |
| Week6 | 6.2 (0.3)  [30] | 8.3 (0.3)  [33] | 2.2 (0.5) | 3.0-1.3 | <.0001 |
| Week7 | 5.8 (0.4)  [29] | 8.0 (0.3)  [36] | 2.1 (0.5) | 3.1-1.2 | <.0001 |
| Week8 | 5.5 (0.4)  [30] | 7.6 (0.4)  [33] | 2.1 (0.6) | 3.3-1.0 | 0.0002 |
| Week9 | 5.1 (0.5)  [30] | 7.3 (0.5)  [34] | 2.2 (0.7) | 3.4-0.8 | 0.002 |

# Abbreviation and explanation: HAM-D_6_=Hamilton Depression Rating Scale, NA=not applicable, SE= standard error, * =baseline for the analysis, CL=confidence limits
