# Supplementary material for: The Day-to-Day Acute Effect of Wake Therapy in Patients with Major Depression Using the HAM-D6 as Primary Outcome Measure: Results from a Randomised Controlled Trial
Source: PLoS One. 2013 Jun 28;8(6):e67264. doi: 10.1371/journal.pone.0067264 (PMC3696105; doi:10.1371/journal.pone.0067264)
Supplement: Table S3 — Results from the sleep logs. (DOC) [file pone.0067264.s005.doc]

**Table S3. Mean values of sleep -onset, -offset, -duration and sleep quality, with standard deviations.**

|  | **Sleep onset** | | **Sleep offset** | | **Sleep duration** | | **Sleep quality** | |
| --- | --- | --- | --- | --- | --- | --- | --- | --- |
| **Day/Intervention** | **Hour: minutes**  **SD** | | **Hour: minutes**  **SD** | | **Hour: minutes**  **SD** | | **Score 0-10**  **(10 = best)**  **SD** | |
| Group | Wake  n=34 | Exercise n=38 | Wake  n=34 | Exercise  n=38 | Wake  n=34 | Exercise  n=38 | Wake  n=34 | Exercise  n=38 |
| Night1 | 23:40 (1:41) | 23:56 (2:09) | 7:21 (1:23) | 7:00 (1:35) | 7:41 (1:44) | 7:04 (1:36) | 5.0 (2.5) | 5.2 (2.4) |
| Night2 (wake I) | NA | 23:29 (1:27) | NA | 6:54 (1:32) | NA | 7:26 (1:54) | NA | 5.9 (2.2) |
| Night3 (recovery sleep I) | 20:31 (0:43) | 23:39 (2:01) | 7:06 (0:54) | 6:56 (1:39) | 10:35 (1:11) | 7:17 (1:20) | 7.6 (2.6) | 5.7 (2.0) |
| Night4 (wake II) | NA | 23:24 (1:03) | NA | 6:51 (1:22) | NA | 7:27 (1:00) | NA | 6.3 (1.6) |
| Night5 (recovery sleep II) | 20:46 (1:21) | 23:32 (1:20) | 7:00 (1:15) | 7:19 (1:37) | 10:14 (1:41) | 7:47 (1:35) | 7.5 (2.5) | 6.2 (1.7) |
| Night6 (wake III) | NA | 23:36 (1:23) | NA | 7:33 (1:35) | NA | 7:57 (1:27) | NA | 6.2 (2.1) |
| Night7 (recovery sleep III) | 21:30 (1:43) | 23:42 (1:18) | 7:21 (1:00) | 6:59 (1:48) | 9:51 (1:58) | 7:17 (1:48) | 7.4 (2.4) | 5.8 (1.9) |
| Night8 | 22:21 (1:08) | 23:16 (1:18) | 6:51 (1:07) | 7:03 (2:03) | 8:30 (1:36) | 7:46 (1:38) | 7.1 (2.5) | 6.4 (2.0) |

Time format is hour: minutes. In all, 35 patients did wake I (sleep data from 34), 34 patients did wake II and 28 patients did wake III (sleep data from 7). Last Observation Carried Forward.
