# Supplementary material for: The Day-to-Day Acute Effect of Wake Therapy in Patients with Major Depression Using the HAM-D6 as Primary Outcome Measure: Results from a Randomised Controlled Trial
Source: PLoS One. 2013 Jun 28;8(6):e67264. doi: 10.1371/journal.pone.0067264 (PMC3696105; doi:10.1371/journal.pone.0067264)
Supplement: Protocol S1 — Study protocol. (DOC) [file pone.0067264.s007.doc]

**Study Protocol**

**of the**

**CHRONOS study**

Is it possible to sustain the antidepressive effect of sleep deprivation in duloxetine-treated patients with major depression by a continued stabilisation of diurnal rhythm and by light treatment?

**
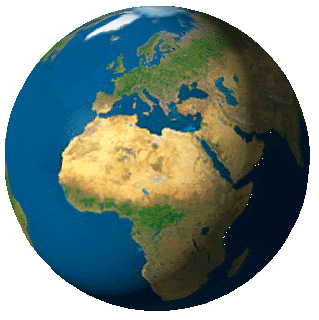
**

Final version

Protocol number 008

EudraCT number: 2005-001855-39

**Table of contents**

1. **Organisation**
   1. Sponsor
   2. Investigator
   3. Financing
   4. Monitor
   5. Data management
   6. Approval
   7. Head of investigation
   8. Authorised staff
   9. Responsible investigator
   10. Other investigators
   11. Project group
   12. Study site
   13. Laws and requirements
2. **Background information**
   1. Antidepressants
   2. Sleep deprivation (wake therapy)
   3. Exercise
3. **Objectives and hypotheses**
4. **Study plan and design**
   1. Study structure
      1. Blinding procedures
      2. Study phases
      3. Medication start-up phase
      4. Hospitalisation period
      5. Follow-up period I
      6. Follow-up period II
      7. Discussion of design
   2. Psychometrics
   3. Efficacy parameters
      1. Primary efficacy parameter
      2. Secondary efficacy parameter
      3. Tertiary efficacy parameter
   4. Randomisation
   5. Medication
      1. Antidepressant medication
      2. Medication for non-psychiatric indications
      3. Medication for psychiatric indications
      4. Medication for patients with bipolar disorder
      5. Drug accountability
   6. Selection of patients
      1. Project information
      2. Inclusion procedure
      3. Inclusion criteria
      4. Exclusion criteria
      5. Discontinuation criteria (end-points)
      6. Protocol deviations
   7. Biochemical and physical tests
5. **Compliance**
6. **Source data**
7. **Patient record**
8. **Patient information and informed consent**
9. **Contacts between the treating medical professionals**
10. **Safety assessment**
11. **Statistics**
12. **Dimensioning**
13. **Time schedule**
14. **Access to source data and other documents pertinent to the study**
15. **Handling and filing of data**
16. **Good Clinical Practice**
17. **Labelling**
18. **Investigator's brochure**
19. **Monitoring**
20. **Ethical concerns**
    1. Change of antidepressant medication
    2. Sleep deprivation
    3. Time of inclusion
21. **Financing and insurance**
22. **Guidelines for publication**
23. **Report**
24. **Copy of study application to producer of study antidepressant**
25. **References**
26. **Enclosures**
27. Summary
28. Approval of the project by the Psychiatric Hospital/Frederiksborg General Hospital
29. Scales (interview scales)
    1. M.I.N.I. (Mini International Neuropsychiatric Interview)
    2. SCID I and II (“Structured Clinical Interview for DSM-IV “)
    3. SPAQ (Seasonal Pattern Assessment Questionnaire)
    4. Hamilton Depression Scale (HAM-D_17)_
    5. Bech-Rafaelsen Melancholia Scale (MES)
    6. Hamilton subscale (HAM-D_6_)
    7. Bech-Rafaelsen Mania Scale (MAS)
    8. Side-effect scale (UKU)
    9. Idiographic questionnaire
    10. Exercise (external assessment scale)
30. Scales (self-rating scales)
    1. Preskorn scale (severity of depression)
    2. Borg scale (exercise)
    3. Morningness-Eveningness-Questionnaire (MEQ)
    4. Symptom Check List (SCL-92)
    5. Scale for expectation to treatment
    6. Scale for experience of treatment
    7. Stanford Sleepiness Scale
31. Diaries
    1. Sleep diary
    2. Light diary
    3. Medication diary
    4. Exercise diary
32. Information to patients
    1. Patient information sheet
    2. Informed consent and acceptance of participation
    3. Information about sleep deprivation
    4. Information about diurnal rhythm (group A)
    5. Instructions for use of light (group A)
    6. Information on collection of 24-hour-urine (group B)
    7. Information on exercise (group B)
33. Information to and from nursing staff
    1. Information about sleep deprivation to staff
    2. The nursing staff's perception of chronobiological intervention (group A)
34. Other Case Report Forms
    1. Medication compliance form
    2. Sociodemographic information
    3. Inclusion and exclusion criteria

**1. ORGANISATION**

**1.1. Sponsor**

Psychiatric Research Unit, Hilleroed, Denmark

**1.2. Investigator**

Psychiatric Research Unit, Hilleroed, Denmark.

**1.3. Financing**

'Unrestricted grant' from Eli Lilly Denmark. The Psychiatric Research Unit thus has sole responsibility for the trial and is both Sponsor and Investigator as far as GCP (Good Clinical Practice) is concerned. Applications for further grants will be made.

**1.4. Monitoring**

The trial will be monitored by the GCP Unit, Copenhagen County Hospital Gentofte, Niels Andersens Vej 65, DK-2900 Hellerup, phone +45 3977-7418.

**1.5. Statistician (data management)**

Ove Aaskoven, Dept. of Clinical Psychology, Odense University Hospital,

phone +45 6541 2040.

**1.6. Approval**

The study has been approved by Mrs. Marianne Wiese, Head of The Psychiatric Hospital, Dyrehavevej 48, DK-3400 Hilleroed (enclosure 2)

**1.7. Head of investigation**

Per Bech, M.D., Professor of Psychiatry, Psychiatric Research Unit, Psychiatric Hospital/Frederiksborg General Hospital, Dyrehavevej 48, DK-3400 Hilleroed, Phone  +45 4829 3318, Fax   +45 4837 3877, e-mail: pebe@fa.dk

**1.8. Authorised staff**

Authorised to sign the protocol and possible later amendments to the protocol:
Per Bech, M.D., Professor of Psychiatry

Klaus Martiny, PhD, specialist in psychiatry, Post-doc.

**1.9. Responsible investigator**

Klaus Martiny, PhD, specialist in psychiatry, Post-doc.

**1.10. Other investigators**

Else Refsgaard, Project Nurse, Psychiatric Research Unit

**1.11. Project group**

Other persons included in the project group:

Jim Bille, Nurse, Co-ordinator of the Quality Assurance Unit, Psychiatric Research Unit, Psychiatric Hospital/Frederiksborg General Hospital, Dyrehavevej 48, DK-3400 Hilleroed

Anne Marie Johansen, Consultant in psychiatry, Dept. 2222, Psychiatric Hospital/Frederiksborg General Hospital, Dyrehavevej 48, DK-3400 Hilleroed.

Anne Dahl, Consultant in psychiatry, Dept. 2221, Psychiatric Hospital/Frederiksborg General Hospital, Dyrehavevej 48, DK-3400 Hilleroed.

Birgit Stråsø, Consultant in psychiatry. Psychiatric outpatient unit Frederikssund, Psychiatric Hospital/Frederiksborg General Hospital, Dyrehavevej 48, DK-3400 Hilleroed.

Jørgen Borup, senior psychiatrist, Dept. 2421, Psychiatric Hospital/Frederiksborg General Hospital, Dyrehavevej 48, DK-3400 Hilleroed.

Vibeke Lund, Physiotherapist, Quality Assurance Unit, Psykiatrisk Sygehus Hillerød, Dyrehavevej 48, 3400 Hillerød.

Lone Borberg, Head Nurse, Psychiatric Hospital/Frederiksborg General Hospital, Dyrehavevej 48, DK-3400 Hilleroed

Peter Treufeldt, Specialist in psychiatry, Medical director, Psychiatric Hospital/Frederiksborg General Hospital, Dyrehavevej 48, DK-3400 Hilleroed

Anna Wirz-Justice, M.D., Professor of Psychiatry, “Centre for Chronobiology”, Basle, Switzerland

Morten Møller, M.D., Professor, Medical-Anatomic Institute, Faculty of Health Science, University of Copenhagen.

Birgitte Welscher, consultant, Head of department, Psychiatric Department, Hvidovre Hospital, Copenhagen.

Ejnar Bundgaard Larsen, consultant in psychiatry, Psychiatric Department, Hvidovre Hospital, Copenhagen.

**1.12. Study site**

Psychiatric Hospital/Frederiksborg General Hospital, Dyrehavevej 48, DK-3400 Hilleroed, Denmark, phone +45 48 29 32 53, fax +45 48 26 38 77.

The Psychiatric Department of Hvidovre Hospital may join the study later and a protocol amendment will be written accordingly and sent to the Danish Medicines Agency and the Scientific Ethics Committee. For this reason the study protocol has been submitted not only to Frederiksborg County, but also to the communities of Frederiksberg and Copenhagen (an entity as far as the Ethics Committee is concerned) for approval.

**1.13. Laws and requirements**

The study will be performed according to the protocol and the valid Danish laws and requirements.

**2. BACKGROUND INFORMATION**

Despite the development of new antidepressants, patients with both unipolar and bipolar depression suffer from continued episodes of depression (Kennedy et al. 2003). New methods are therefore asked for to bring these episodes to an end within a short time (Kessing et al. 2004, Bech et al. 2005) in order to reduce the period of time in which the patients are depressed and the number of suicides

(Sokero et al 2005). In the following the various elements pertinent to the investigation will be described.

**2.1. Antidepressants**

All patients will receive the antidepressant duloxetine. Duloxetine has recently been approved by the Danish Medicines Agency. Among the drugs having an effect on both types of transmitters (SNRIs) this drug has been selected because it has a balanced re-uptake inhibition of both serotonin and noradrenalin. In regard to effect, duloxetine is related to venlafaxine which also is an SNRI. Venlafaxine, which is used frequently in the treatment of hospitalised patients with depression, requires a fairly high dosage before the noradrenalin reuptake inhibition starts operating. In the dose range of 40 to 120 mg/daily, duloxetine has shown a dose-response correlation with a minimal effective dose of 60 mg.

**2.2. Sleep deprivation (wake therapy)**

Sleep deprivation is a well-known method, which in numerous studies has proved its ability to eliminate or reduce the severity of the depression within just a few hours (Ostenfeld 1973, Ostenfeld 1986, Kuhs and Tölle 1991, Smeraldi 1999, Benedetti 2001). Several Danish studies have confirmed this effect (Svendsen 1976, Larsen et al., 1976, Kvist et al. 1980). Sleep deprivation can be carried out in several ways. In partial sleep deprivation the patient is waked up at 2.30 a.m. In the following the patient has to stay awake for the rest of the night and all next day and is not allowed to go to sleep again until the following night (Schilgen et al. 1987). In total sleep deprivation, the patient is awake all night and the following day. The most recent sleep deprivation regimens require that the patient goes through 3 partial or total sleep deprivations in the course of one week, but always with one regular night of sleep to follow one night of sleep deprivation.

In older days, sleep deprivation was quite well-known and senior nursing staff and doctors can describe how many institutions used sleep deprivation in the treatment of depression. We don't know precisely why the method has been abandoned, but it is only used sporadically in Denmark nowadays. The method is used routinely in a number of countries, among them Germany, Austria and several locations in the USA. The development of pharmacological treatment methods in beginning of the 1950s and faith in the effect of these new drugs is probably one of the reasons for the abandonment of sleep deprivation. However, a continued interest in sleep deprivation can be documented by the large number of publications about the method. When we made a search National Library of Medicine’s ”PubMed" in the beginning of 2005, the number of titles referring to depression and sleep deprivation was 622. 112 of these had appeared after the year 2000, and 20 had been published within the last calendar year.

The essence of the published results is that approximately 60-70% of all patients with major depression respond well to one night's sleep deprivation, i.e. the degree of their depression is reduced by 50% or more (response), and a number of patients will even be without any symptoms of depression (remission) (Wirz-Justice et al. 1999, Wirz-Justice et al. 2004).

Patients whose depression is part of a bipolar illness often experience a better effect from sleep deprivation than patients whose depression is part of a unipolar depression. Clinical experience has shown that bipolar as well as unipolar patients without significant personality disorders and patients with large day-to-day variations within their depressive condition achieve the best treatment results. Bipolar as well as unipolar depression fulfilling these criteria (no significant personality disorder or large variations) experience an equal gain from both SSRIs, SNRIs (Goodwin 2003) and lithium (Bech et al. 1980).

Patients who experience a large day-to-day variation in depression severity have a serious social handicap, because planning ahead – activities and work - is very difficult for them due to their unstable condition. On average, patients with bipolar depression do not only experience a larger number of episodes than those with unipolar depression (Angst et al. 2003), but also a continued risk of new episodes, maybe even an increasing risk as they grow older (Kessing et al. 2004, Keller 2004, Bech et al. 2005). These patients are therefore natural targets for new treatment methods. Sleep deprivation and the day structure they are asked to maintain afterwards hopefully can be an instrument they can use on their own initiative to fight an illness which is recurrent for many of them.

Mostly (but not always), the effect of one night's sleep deprivation is short, and most patients will be back in their depressive condition after the following night's sleep. To overcome the brevity of the effect and the relapse to the previous condition, several methods have been developed:

- The simplest method is repeated sleep deprivation a number of times, resulting in a continued effect and an increased duration. Most centres use 3-6 sleep deprivations. This increased effect by repetition is well-known from other treatments, e.g. ECT (Electro Convulsive Treatment).
- Light therapy is used at the end of the sleep deprivation night (for 30 minutes at 4.00 a.m.), partly to reduce tiredness (Colombo et al. 2000, Benedetti et al. 2001, Fritzsche et al. 2001, Neumeister et al. 1996) and partly as a daily therapy at mornings, possibly as long-term treatment, thus implying that the patient continues with it after discharge from hospital (Martiny 2004, Benedetti et al. 2003). Light therapy is used irrespective of season.

Sleep-phase-advance, in which the patient goes to sleep early and is woken-up early, i.e. the patient's diurnal rhythm is "advanced" compared to the usual pattern of sleep. Several studies have shown that ”sleep-phase-advance” in itself has had an effect similar to partial or complete sleep deprivation (Voderholzer 2003, Berger 2003). In this study, sleep-phase-advance is used in a version modified according to the possibilities of a psychiatric ward and where the aim is to maintain the improvement that has been obtained by sleep deprivation.

- Trials with pindolol (β-blocker usually used to treat high blood pressure) as an addition to sleep deprivation have shown that patients treated with pindolol experienced a better antidepressant effect and fewer relapses than the control group which solely received sleep deprivation (Smeraldi et al., 1999).
- Trials with lithium (mood stabiliser) showed that this drug was able to enhance and maintain the effect of sleep deprivation (Szuba et al. 1994, Benedetti et al. 2001).

Sleep deprivation must always be followed by a night of normal sleep (recovery sleep). Patients receiving sleep deprivation are usually hospitalised, but some patients use the method at home after sleep deprivation at a hospital or clinic (Loving et al. 2002).

The risk of switching into a manic episode after sleep deprivation is of the same magnitude as for antidepressants (Colombo 1999). No excessive frequency of suicide has been seen after its use (Vovin Ria et al. 1985, Kuhs et al. 1991).

Clinical experiences with sleep deprivation show a lower effect in patients who have not responded to long-term pharmacological treatment with several antidepressants with varying mechanisms of action (refractory depressions).

The great advantage of sleep deprivation is that it can remove depression within a few days. Together with additive treatment with duloxetine, light and a regulation of diurnal rhythm (sleep phase advance) it appears that a stabilisation of improvement can be obtained. Nothing, however, points toward a lifelong effect of the treatment, and it seems unclear whether the method removes the current depressive phase implies that you "skip" a depression or that you postpone it. It is outside the scope of this study to assess long-term effects of this treatment, as the study period is limited to 29 weeks. A study period of 29 weeks has been chosen, because internationally you distinguish between relapse (relapsing into the current episode of illness) and recurrence (occurrence of a new episode) after 6 months. In the present study the patients are thus followed over a period exceeding the expected duration of a depressive episode. Patients who by means of combined treatment (sleep deprivation, duloxetine, light and maintenance of diurnal rhythm) get out of their depression and have not remitted within 6 months will thus, according to this definition, be said to have been treated successfully for the current period of time.

**2.3. Exercise**

Exercise has shown to have an impact on the diurnal rhythm of healthy study subjects (Buxton et al. 2003) and several randomized trials of the effect of exercise on depression are currently ongoing. It is expected that the effect of exercise on depression has a slower onset of action than the effect of sleep deprivation (Blumenthal et 1999, Babyak et al 2000) and that there will be a connection between the degree of exercise (intensity and stamina) and the development of the degree of depression. Therefore, exercise is a good alternative to sleep deprivation and has therefore been chosen as part of the treatment in the non-sleep deprivation group (group B). The exercise programme is to be commenced during hospitalisation. The intensity of the exercise is adapted to the single patient, but 30 minutes daily are aimed at, as recommended in the 2004 campaign by the National Health Board (Sundhedsstyrelsen 2004). The patients continue doing their exercises throughout the study period and compliance is monitored at each visit by means of interview forms and exercise diaries.

**3. OBJECTIVE**

The main objective of the trials is to examine whether sleep deprivation in combination with light treatment, duloxetine and maintenance of diurnal rhythm (Group A) is able to induce a faster onset of action and lasting antidepressive response compared to an individually designed exercise programme in combination with treatment with duloxetine (Group B).

Secondary objectives are studies of cortisol concentration, variability of the degree of depression, the idiographic method and finally, applicability and effect of exercise over 9 weeks under supervision. The following hypotheses ar going to be tested in the study:

- Is sleep deprivation in combination with duloxetine, light and maintenance of diurnal rhythm (Group A) able to induce a greater degree of response or remission than exercise in combination with duloxetine (Group B). The hypothesis is tested after 2, 9 and 29 weeks of therapy?
- Is it possible for patients with major depression during the supervised course of treatment of 9 weeks to achieve the National Health Board's recommendation of exercising 30 minutes per day, and is there any association between the exercise effort and the degree of depression after 29 weeks?
- Is the cortisol concentration as measured in urine and saliva the beginning of week 2 correlated to the severity of depression as measured after 2, 9 and 29 weeks of therapy?
- Is the concentration of cortisol measured in saliva correlated with the concentration of cortisol in diurnal urine?
- Is the idiographic method more sensitive to change of degree of depression than clinician- and patient-rated scales and questionnaires for depression?
- Can variability in the degree of depression as measured with the Preskorn scale (see psychometrics) predict response to sleep deprivation?

**4. STUDY PLAN AND DESIGN**

**4.1. Study structure**

The study examines the difference between antidepressant effect of two different chronobiological principles in patients with major depression treated with duloxetine (A and B). The trial has the design of a controlled clinical trial, in which the patients are randomised to either one of the two treatment groups (A or B). The study is single-blind, as the rater of the severity degree of depression (Hamilton scale) is blinded towards the treatment group to which the interviewed patient belongs.

4.1.1. Blinding procedures

It is not possible to blind the patient towards type of treatment (sleep deprivation or exercise). It is, however, possible to blind a permanent rater towards the treatment the patient has received. This can be done by having a rater who interviews and rates the patient each time throughout the whole study period by means of the Hamilton scale. The patients will be informed that they are not to disclose the type of treatment they receive to this permanent rater who assesses severity of depression according to Hamilton. There is no item in the Hamilton scale to reveal the type of treatment the patient receives, not even the sleep item, as all patients record their sleep in a sleep diary. The rater who conducts the interview will not meet the patient in the ward, as the regular visits are outside (before and after) the period of hospitalisation and will not be permitted to see patient records or CRFs. This rater will only receive the Hamilton rating sheet which does not contain information as to treatment group. All other procedures of the study will be performed by unblinded evaluators.

4.1.2. Study phases

The number of visits is equal in the two groups. See flowcharts 1 and 2 and Figures 1 and 2 on the following pages.

The study consists of 4 phases, the contents of which are described in detail in the following.

4.1.3. Medication start phase**:** week 0 (7 days)

*Groups A and B*

After the patient has signed the informed consent form, an agreement concerning overnight stay is made with an in-patient ward in one of the county's psychiatric wards. When this has been decided, the patient can be included in the study and randomised to group A or B. All patients are switched from their present antidepressant treatment to duloxetine 60 mg daily in the morning. Patients who do not receive any current pharmacological treatment will also receive duloxetine 60 mg daily. Patient data are collected by means of questionnaires and forms as shown in flowchart 1 under inclusion visit. A deviation of +/- 2 days (window) of the duration of this phase due to a possible acute shortage of beds in the in-patient ward is acceptable.

Group B

At inclusion this group is also elucidated idiographically in regard to depression symptoms.

4.1.4. Hospitalisation phase: week 1 (7 days)

All study participants are hospitalised and meetings with the study investigators are envisaged for each working day. All patients (both groups, A and B) are hospitalised in an open in-patient ward. See Figures 1 and 2. Previously hospitalised patients can be discharged if their condition permits it. Patients admitted due to study participation will be discharged 6 days after admission unless their condition does not allow it. If the patient stays in hospital the trial will follow the usual guidelines, but the visits will take place in the in-patient ward whenever necessary.

Group A

- During hospitalisation, Group A patients go through 3 sleep deprivations, the first one on the day of admission (see Figure 2). Sleep deprivation is mainly carried out as total sleep deprivation, i.e. the patients stay awake all night. However, in some cases partial sleep deprivation may be used, in which the patient is woken up at 02.00 a.m. and has to stay awake for the remains of the night. This can be done either due to an individual patient evaluation made by the investigator or due to a wish on the part of the patient. There may be good reason for using partial sleep deprivation in case of medical diseases such as diabetes, where the blood sugar level can get out of balance, or in other diseases that may be influenced by a longer wake period. Also elderly patients expect not to be able to stay awake all night may also profit from partial sleep deprivation. Patients who experience that their first and total sleep deprivation is too demanding may also switch over to partial sleep deprivation. A combination of total and partial sleep deprivation is thus permitted.
- In the evening after each sleep deprivation the patients must go to bed at 9 p.m. at the very latest and get up at 7 a.m. the following morning (sleep-phase advance).
- The treatment with duloxetine continues unchanged, with a dose of 60 mg daily.
- The patients are informed about light treatment on the day of admission which is given for the first time the following morning and subsequently each day of the study period. The duration of light treatment is 30 minutes. The time of day of the light treatment is decided after completion of the MEQ questionnaire (see psychometrics paragraph). In addition to this, to fight tiredness, the patients take light treatment at 4 a.m. each night during the period of sleep deprivation.
- At each visit, the patients are taught how to maintain their diurnal rhythm after discharge by an approximate bed time at 11 p.m. and a getting-up time at 8 a.m. the very latest. The patients receive instructions about the use of the medication and sleep diaries.
- Apart from exercise, the patients are allowed to join all milieu-therapeutic activities in the ward.

Group B

- In one of the first few days after admission, Group B patients are instructed by a physiotherapist in an individually designed exercise programme. A plan for follow-up meetings with the physiotherapist is agreed upon. The number of follow-up meetings is the same as the number of visits to the study clinicians who assess treatment effect, and if possible all planned visits during the study will be scheduled together. The patients are allowed to join all milieu-therapeutic activities in the ward.
- Duloxetine treatment continues unchanged, with a dose of 60 mg daily.
- During the first 24 hours in the hospital the patients collect 24-hour urine for the measurement of cortisol. On their second day the patients give saliva samples in the morning for the measurement of cortisol.

4.1.5. Follow-up period I: week 2 until week 9 (included) (7 weeks)

During this phase the patients are examined weekly.

Group A

- Patients with scores of 8 or more on the Hamilton Depression Scale at visit 4, 5 or 6 will be offered two additional – either total or partial - sleep deprivations in the week of their visit. If practical reasons prevent this, the two sleep deprivations can be performed in the following week. Both sleep deprivations must be performed during the same week (preferably Tuesday and Thursday). The patients should be admitted in the evening, preferably Tuesday for the first and Thursday for the second sleep deprivation. The patients stay awake during the night in the ward and can be discharged next morning. No further sleep deprivations are permitted during the remaining study period and they are not allowed to carry out sleep deprivation on their own at home at any time.
- Duloxetine treatment is continued with an unchanged daily dose of 60 mg.
- Light treatment at home is continued, with daily treatments as in the previous study phase. The patients can borrow the lamp from the hospital.
- Patient education concerning maintenance of diurnal rhythm is on a continuous basis, at each visit, with an envisaged bedtime at 11 PM and a wake-up time at 8 AM at the very latest.
- The patients are instructed on a continuous basis how to fill in medication diary, light diary and sleep diary.

Group B

- Duloxetine treatment is continued with an unchanged daily dose of 60 mg.
- The patients receive weekly instructions and training within their individual exercise program. The time at which the patients have to show up should as far as possible be coordinated with the other visits to the hospital.

4.1.6. Follow-up period II: week 10 to week 29 (incl.) (20 weeks)

The patients are seen every 4th week.

Duloxetine treatment is continued with an unchanged daily dose, with a possibility to increase the dose to a maximum of 120 mg daily when according to a clinical evaluation the patient's condition has not improved. When side-effects are seen or if there is a lack of effect even after increasing the duloxetine dose to maximum, it is permitted to change to another antidepressant.

- Light treatment at home is continued as in the previous study phase.
- At the planned visits each 4th week the patients are instructed on a continuous basis how to maintain their diurnal rhythm, aiming at a bedtime at 11 PM and a wake-up time at 8 AM at the very latest.
- The patients are instructed on a continuous basis how to fill in medication diary, light diary and sleep diary.

Group B

- Duloxetine treatment is continued with an unchanged daily dose of 60 mg.
- The patients receive weekly instructions and training within their individual exercise program. The time at which the patients have to show up should as far as possible be coordinated with the time for other visits to the hospital.
- Duloxetine treatment is continued with an unchanged daily dose, with a possibility to increase the dose to a maximum of 120 mg daily when according to a clinical evaluation the patient's condition has not improved. When side-effects are seen or if there is a lack of effect even after increasing the duloxetine dose to maximum, it is permitted to switch to another antidepressant.
- Every 4 weeks the patients receive instructions and training within their individual exercise programme. The time at which the patients have to show up should as far as possible be identical with the other visits to the hospital.

4.1.7. Discussion of design

The choice of a single-blinded trial was the best choice of design, as it is not possible to blind the study towards the patients. To secure placebo response in both groups, this design was chosen to encourage the same expectations as to effect of treatment in both treatment groups. In both treatment groups the used methods aim at a change or stabilisation of diurnal rhythm. Exercise as well as sleep deprivation function as "zeitgebers" (time givers ). We thus look at two different chronotherapeutic interventions. It can be expected that sleep deprivation will have an immediate effect, while the effect of exercise is seen only after a certain period of time. In follow-up phase I, group B will thus function as a control group, while follow-up phase II will be a comparison of two different methods.

Exercise has also been chosen, because it is a very well-accepted treatment which in the same way as light treatment and sleep deprivation makes it possible for the patients to make an effort to improve their own condition. The individual adaptation of the exercise programme ensures that the demands are not too high and thereby act a demotivation. An idiographic examination is only made in group B. The reason for this is to ensure an even weighting of expectations from the two groups. Cortisol measurements in saliva and urine are made during hospitalisation and only in group B patients in order not to overstretch sleep deprivation patients. The medication start period has been chosen, so patients don't experience possible new side effects and exercise/sleep deprivation at the same time. It is also an advantage that duloxetine has reached a steady state concentration in the body before the other interventions show an effect.

The steady state of duloxetine concentrations is also an advantage in relation to the effects of the other interventions.

Group A is flexible as to number of sleep deprivations, with the possibility of administering two additional sleep deprivations in weeks 4, 5 or 6. This is to give patients with severe depression a better chance of remission without inconveniencing patients with less severe depressions to no avail.

The choice between total and partial sleep deprivation has been introduced to be able to adjust the strain to the patients' condition. By this measure we take a step towards clinical reality, to which we aim to generalise the results of the study.

| **Study period and duration** | **Medication start phase:**  **7 days** | **Hospitalisation:**  **7 days**  Visits on week days | **Follow-up phase I:**  **7 weeks**  **Visits each week** | | | | | | | |
| --- | --- | --- | --- | --- | --- | --- | --- | --- | --- | --- |
| **Week no.** | **0** | **1** | **2** | **3** | **4** | **5** | **6** | **7** | **8** | **9** |
| **Visit no.** | **1 (Inclusion)** | **2** | **3** | **4** | **5** | **6** | **7** | **8** | **9** | **10** |
| **M.I.N.I.+ SCID I and II**  **SPAQ + MEQ + DIAD + SPS** | x |  |  |  |  |  |  |  |  |  |
| **Hamilton + MES + MAS + MDI + UKU + WHO-5** | x | x | x | x | x | x | x | x | x | x |
| **Preskorn** | daily | daily | daily | daily | daily | daily | daily | daily | daily | daily |
| **Borg scale + external assessment (exercise)** |  | x | x | x | x | x | x | x | x | x |
| **SCL-92** | x | x | x | x | x | x | x | x | x | x |
| **Idiographic method (gr. B)** | x | x | x | x | x | x | x | x | x | x |
| **EKG + Blood pressure + Blood samples** | x |  |  |  |  |  |  |  |  | x |
| **Collecting diurnal urine (gr. B)** |  | x |  |  |  |  |  |  |  |  |
| **Saliva samples (gr. B)** |  | x |  |  |  |  |  |  |  |  |
| **Sleep, light, medication and exercise diaries and status** | x | x | x | x | x | x | x | x | x | x |
| **Stanford Sleepiness Scale** |  | x |  |  |  |  |  |  |  |  |
| **Expectation to treatment** | x |  |  |  |  |  |  |  |  |  |
| **Evaluation of treatment** |  |  | x |  |  |  |  |  |  | x |
| **TEMPS-A** |  |  |  |  |  |  |  |  |  |  |

**Flowchart 1(week 0 to 9, duration 9 weeks**)

| **Study period and duration** | **Follow-up phase II:**  **20 weeks**  **Visit every 4th week** | | | | | |
| --- | --- | --- | --- | --- | --- | --- |
| **Week no.** | **9*** | **13** | **17** | **21** | **25** | **29** |
| **Visit no.** | **10** | **11** | **12** | **13** | **14** | **15** |
| **M.I.N.I.+ SCID I and II**  **SPAQ + MEQ + DIAD + SPS** |  |  |  |  |  |  |
| **Hamilton + MES + MAS + MDI + UKU + WHO-5** | x | x | x | x | x | x |
| **Preskorn** | weekly | weekly | weekly | weekly | weekly | weekly |
| **Borg scale + external assessment (exercise)** | x | x | x | x | x | x |
| **SCL-92** | x | x | x | x | x | x |
| **Idiographic method (gr. B)** | x |  |  |  |  | x |
| **EKG + Blood pressure + Blood samples** | x |  |  |  |  | x |
| **Collecting diurnal urine (gr. B)** | x |  |  |  |  | x |
| **Saliva samples (gr. B)** |  |  |  |  |  |  |
| **Sleep, light, medication and exercise diaries and status** | x | x | x | x | x | x |
| **Stanford Sleepiness Scale** |  |  |  |  |  |  |
| **Expectation to treatment** |  |  |  |  |  |  |
| **Evaluation of treatment** | x |  |  |  |  | x |
| **TEMPS-A** |  |  |  |  |  | x |
| **WHO-Five** | x | x | x | x | x | x |

**Flowchart 2 (week 9 to 29, duration 20 weeks)**

***Week 9 is a repetition of flowchart 1 to clarify the trial phases**

**Figure 1. Duration of treatment phases and the attached treatment elements**

| **Day visit** | **Hospitalisation** | **Day visit** | **Day visit** |
| --- | --- | --- | --- |
| **Duloxetine start** | **Sleep deprivation + light + sleep-phase-advance / exercise** | **Diurnal rhythm + light+ additional sleep deprivations in week 4, 5 or 6 / exercise** | **Diurnal rhythm + light/ exercise** |

| **Medication start phase** | **Hospitalisation phase** | **Follow-up phase I** | **Follow-up phase II** |
| --- | --- | --- | --- |
| **1 week** | **1 week** | **7 weeks** | **20 weeks** |

**Figure 2. Example of the course of sleep deprivation and sleep-phase-advance (SPA) with admission on a Monday and discharge on a Saturday.**

| **Sleep deprivation I** | **SPA I** | **Sleep deprivation II** | **SPA II** | **Sleep deprivation III** | **SPA III** |
| --- | --- | --- | --- | --- | --- |

| **Admission** |  |  |  |  | **Discharge** |  |
| --- | --- | --- | --- | --- | --- | --- |

| **Monday** | **Tuesday** | **Wednesday** | **Thursday** | **Friday** | **Saturday** | **Sunday** |
| --- | --- | --- | --- | --- | --- | --- |

**4.2. Psychometrics**

To make a diagnosis, to examine co-morbidity and possible exclusion diagnoses the M.I.N.I. (Mini International Neuropsychiatric Interview) (Sheehan et al. 1998) and the SCID (First, 2002) will used, both based on the American DSM-IV diagnostic system (APA 1994). DSM-IV also contains the ”Seasonal Pattern Specifier” we use for the diagnosis of winter depression (”Seasonal Affective Disorder”). To elucidate atypical depression, the DIAD (Diagnostic Interview for Atypical Depression) (Terman et al 2003) is used.

Patients with personality disorders are identified and excluded by means of the SCID (First, 2002). DSM-IV subclassifies major depression into ”Major depressive disorder, single episode” (296.2) and ”Major depressive disorder, recurrent” (296.3). Bipolar disorder is first classified by using the M.I.N.I. criteria for ”previous hypomanic/manic episode”, and patients fulfilling the diagnostic criteria for previous manic or hypomanic episode are then subdivided according to DSM-IV into the diagnostic codes ”Bipolar I disorder, most recent episode depressed” (296.5), ”Bipolar I disorder, most recent episode unspecified” (296.7), and "Bipolar II disorder" (296.89).

For the assessment of severity of depression clinician-rated as well as self-rating scales are used.

The clinician-rated scales are the 17-item Hamilton Depression Scale (HAM-D_17_), (Bech et al. 1986), the Melancholia Scale (MES) (Bech 2002a), and a Hamilton Depression 6-item subscale (HAM-D_6_), (O'Sullivan et al. 1997, Licht et al. 2005). Sleep studies usually use the HAM-D_6_, as it has no sleep item. During hospitalisation, the patients are assessed daily with the HAM-D_6_. During hospitalisation, patient assessments are made daily with HAM-D_6_. A minimum score of 13 on the 17-item Hamilton Depression Scale has been chosen for inclusion, as some patients with long term depressions have scores on this moderate level, which, however, has a marked impact on social functioning and quality of life. This is owing to the peripheral depression symptoms which still are present. Another reason is that one single measurement may be unreliable in patients with great day-to-day variability of the degree of depression. It has also been demonstrated that the effect of antidepressant treatment starts at this level (Paykel 1990).

The self-rating scales used are the SCL-92 (Symptom Checklist) (Olsen et al 2004) and the Major Depression Inventory (MDI) (Bech et al. 2001, Olsen et al. 2003), - the MDI, however, in a modified version in which severity as well as frequency of the depression symptoms are assessed .

To subjectively assess the day-to-day and time of day variability of the degree of depression, the Preskorn scale will be used daily at 9 and 12 a.m. and 3, 6 and 9 p.m., and at awakening and bedtime during the first week prior to hospitalisation. It will be examined whether variability of degree of depression and diurnal variation are predictors of response to sleep deprivation. During the first 9 weeks of the study, the Preskorn questionnaire will also be used for daily measurements of degree of depression, and subsequently once weekly until week 29.

A questionnaire has been developed for the patient's assessment of his/her expectation to treatment, and thus an evaluation of an even distribution of the placebo effect among the two treatment groups. It is filled in at inclusion, after the patient has received detailed information about the project.

The WHO-Five Well-being Index (WHO-5) will be used at each visit to assess quality of life. This instrument contains 5 questions and has been validated in a number of studies (Bech 2004).

Specific questionnaires have been developed to capture the patient's impression of the treatment after 2 weeks (i.e. immediately after discharge), also offering the possibility of open statements. After 9 and 29 weeks, the patients are asked for repeated assessments of their treatment.

The patient's temperament is assessed by the TEMPS-A at the end of the study, in week 29 (Akiskal et al. 2002). This questionnaire contains five scales examining personality traits that have been present throughout the patients' lives (character traits). The five traits are: depressiveness ("depressive"), anxiousness ("anxiety"), irritability ("irritable"), mood swings ("cyclothymia") and extraversion, energeticness ("hyperthymia"). The reason for using this scale at the end of the study is that the depressive condition, which expectedly is less marked at the end of the study, at an earlier stage would affect the self-assessment of the person and thus rather reflect the current condition than character traits.

To decide at which hour light treatment should be administered, the Morningness-Eveningness Questionnaire (MEQ) will be used (Horne et al. 1976, Horne et al. 1977, Terman et al 2005).

Side effects will be rated by means of the UKU (Udvalget for Kliniske Undersøgelser, evaluation form for side effects) (Lingjærde et al. 1987), with relevant questions concerning serotonergic and noradrenergic side effects.

The Mania Scale (MAS) is to be used at all visits to assess the possible development of a hypomanic or manic condition. A MAS score of 15 or more thus means exclusion from the study.

The SPAQ (Seasonal Pattern Assessment Questionnaire) (Kasper et al 1989) questionnaire will be used to assess seasonal variability of the depression.

The idiographic method is to be used at all visits, but only in group B patients. The method implies free constructs by the patients who describe the symptoms they have experienced in connection with their current depressive episode. At each visit, the free construct is given as a percentage value in relation to the baseline construct (set 100% for each construct at baseline, e.g. how much of 100% tiredness is your tiredness today?), thus measuring change from inclusion an onwards. In contrast to the other scales used in the study, this method is not an absolute measure of severity. With this method we hope to obtain a more balanced picture of the symptoms we obtain by standardised methods such as questionnaires and rating scales, and we also hope to accomplish a greater degree of sensitivity towards change in the condition. The strength of this method is that symptoms which patients have experienced during their depression are not forced into the standardised boundaries of a rating scale or a questionnaire. The method is based on Kelly's ideas on free constructs (Kelly, 1955). It has been used in GRID-QOL (Thunedborg et al 1995), a computer-assisted quality of life rating system.

At inclusion, sleep and exercise are evaluated for both groups. Sleep diaries, exercise diaries and medication diaries are completed in both groups at each visit. Light diaries are only completed in group A.

The scales are employed as indicated in flowcharts 1 and 2. Flowchart 1 covers the first 9 weeks while flowchart 2 covers weeks 10 to 29.

**4.3. Efficacy parameters**

4.3.1 The primary efficacy parameter is the number of patients in the two group that have achieved response to treatment or remission after 2, 9 or 29 weeks. Response is defined as a more than 50% reduction in HAM-D_17_ scores after inclusion into the study while remission is defined as a score of 7 or less on the HAM-D_17_.

4.3.2. Secondary effect parameter is the number of those patients in the two treatment groups who have experienced long-lasting response or remission after 9 weeks and after 29 weeks. Lasting response is defined as patients responding after 2 weeks of enrolment in the study and the response either lasting until week 9 (early lasting response) or until week 29 (episode response). Lasting remission is defined as being in remission after 2 weeks in the study and the remission either lasting until week 9 (early lasting remission) or until week 29 (episode remission). Response and remission are defined as mentioned under primary efficacy parameter. Patients who according to the protocol receive further sleep deprivations in week 4, 5, or 6 thus are cannot be defined as being in early, lasting remission. Response and emission are defined as mentioned under primary efficacy parameter. Patients who according to the protocol carry out additional sleep deprivation in weeks 4, 5 or 6 thus are defined not to be in early lasting remission.

4.3.3 Tertiary effect parameters are

- The extent of exercise in follow-up periods I and II, measured as a combined assessment of the exercise effort by the Borg scale and an external evaluation.
- Cortisol concentration at inclusion (in saliva or diurnal urine) compared with cortisol after 2, 9 and 29 weeks.
- The cortisol concentration in diurnal urine at inclusion is compared with the cortisol concentration in saliva at inclusion.
- The percentage of variability of depression severity at week 0 (calculated from the Preskorn scores during the first 6 days of the study) is compared to change in depression severity after the 3 sleep deprivations at week 2.
- In treatment group B, the idiographic effect parameter is the percentage change from inclusion to week 29.

**4.4. Randomisation**

A computer-generated program for the randomisation codes was developed by the study statistician, Mr. Ove Aaskoven, Department of Clinical Psychology, Odense University Hospital. The randomisation codes are in blocks. The number of patients per block is determined by the statistician and blinded towards the investigators. Each patient receives a numbered envelope prepared by the statistician and a secretary (Ms. Lone Lindberg), containing a letter stating one of the two treatment groups. The envelopes are numbered and distributed consecutively, i.e. patient 1 gets the envelope with the number 1, patient 2 the envelope with number 2 etc. The patients, who after having received oral and written information have given their written informed consent to be included in the study, receive and open the envelope with information about the group they have been randomised to. The envelopes are kept in a locked closet at the Research Unit and are handed over to the investigator upon presentation of the signed informed consent statement on the day of inclusion. As the study is blinded towards the Hamilton rater, this rater will have no access to study patient records or study CRFs, in which the treatment group (A or B) is registered.

**4.5. Medication**

4.5.1. Antidepressants

The medication used in the study is duloxetine, an SSRI (selective serotonin reuptake inhibitor) that acts by inhibiting reuptake of serotonin and noradrenalin into the brain. Duloxetine is a drug registered for the indication "major depression". The patients are switched from any other drug they may use up to the time of inclusion to duloxetine. Thus, there is no transition phase and a study patient is treated with duloxetine from the first day in the study. This is the reason why patients treated with marplan (isocarboxazide) cannot enter the study. Patients treated with fluoxetine need to take a 1-week's break before being enrolled. Duloxetine was chosen because it is expected to have a positive side-effect profile and a proven antidepressant effect (Detke et al. 2002, Raskin et al. 2003). Duloxetine is administered in a fixed dose of 60mg daily. In specific cases, this dose may be reduced during the first 9 weeks, taking account of side effects. If there is no clinical effect after the first 9 weeks, the daily dose may be increased to 120mg. In case of lacking effect or of side effects, another antidepressant may be administered. No other antidepressants must be given together with duloxetine. Switching to another antidepressant is no reason for exclusion from the study (see "Criteria for exclusion from the study"). The duloxetine doses are described in detail in the description of the trial phases in the trial design chapter.

4.5.2. Medication for non-psychiatric indications (physical illness)

This kind of medication can be used as prescribed, provided it has no significant clinical interactions with duloxetine. Medication prescribed by other doctors during the study may be used if it doesn't interact with duloxetine or any other medication the patient is taking.

4.5.3. Medication for other psychiatric indications

This includes anxiolytics (anti-anxiety drugs) and hypnotics (drugs against sleeplessness). Current treatment with hypnotics and anxiolytics can continue unchanged throughout the whole study period, with necessary dose adaptations according to recommendation and condition. An increase of anxiolytics and hypnotics is permitted throughout the whole study period. The following new treatments are permitted during the study:

- Oxazepam in daily doses of up to 45 mg for severe anxiety
- Zopiclone in daily doses of 7.5 mg for sleep disturbances

4.5.4. Medication for bipolar disorder

Patients with bipolar depression must be in treatment with a mood stabilizer such as lithium, carbamazepine, lamotrigine, or valproate according to recommendations and in recommended doses before they enter the study. Patients with bipolar disorder, who do not receive mood-stabilising treatment, can enter the study as soon as they fulfil the conditions mentioned above. These requirements, however, must not lead to keeping patients from receiving other, optimal depression treatment. It is not allowed to use antipsychotics, as these may inhibit the effect of sleep deprivation.

4.5.5. Drug accountability

All drugs, used or returned, are accounted for, and a drug registration log is kept in which this is registered at each visit. Surplus medication will be disposed of by the hospital dispensary. All information about other medication the patient uses will be registered in a special medication form throughout the whole study period. The types of mood stabilizers for patients with bipolar disorders cannot be changed, but the dosage may be adjusted if necessary. Patients who according to clinical evaluation have to be switched to another medicaton, must discontinue the study (see "Critieria for exclusion from the study"). If serum monitoring (e.g. lithium or lamotrigine) is required, this will be done according to general clinical practice. During hospitalisation, the medication is administered by the nursing staff.

**4.6. Selection of patients**

4.6.1. Information about the project

All patients examined at the Psychiatric Research Unit of Frederiksborg County are from Frederiksborg County while patients examined at Hvidovre Hospital will be from those parts of Copenhagen that constitute the Hvidovre Department of Psychiatry's catchment area (Kongens Enghave, Vesterbro, Vanløse and Valby). Accordingly, the project has to be approved by the Research Ethics Committees of Frederiksborg County and the Copenhagen and Frederiksborg communities.

Hospitalised patients as well as day patients can be included in the project. The Psychiatric Research Unit in Hilleroed (Frederiksborg County) has performed clinical trials within the area of depression for tha last many years and has thus established a good cooperation with GPs and psychiatric specialists in psychiatric practices in Frederiksborg County. Furthermore, the unit has good relations to all wards of the Frederiksborg County Department of Psychiatry's local subdepartments in the region (Frederikssund, Elsinore, Hoersholm and Hilleroed). The Head of the Department of Psychiatry of the county supports the project by joining the project group and making available the necessary beds. The Psychiatric Research Unit has distributed information about the project to doctors and nurses at the Frederiksborg County Department of Psychiatry. All wards and day units are informed about the project.

To recruit possible participants to the project, all GPs, psychiatric specialist practices and local sub-departments of psychiatry (wards and day units) will receive written information about the study. The investigators will keep informed about admissions of new patients who might be suitable for and interested in participation in the study. All psychiatric wards will be contacted weekly. At Hvidovre Hospital, the investigators will keep informed about admissions of new patients who might be suitable for and interested in later participation in the study. However, to secure the best possible project start, the study will commence at the Frederiksborg County Department of Psychiatry at Hilleroed.

The study design implies that study participants must make an effort on their own, either by doing exercises or by being treated with a combination of light, sleep deprivation and maintenance of diurnal rhythm. We expect that many patients will be interested in participating in a study using new methods of treatment, but also that patients with severe depression may have trouble being in the project. This has been taken into consideration in the inclusion criteria and in the written patient information.

4.6.2. Inclusion procedure

Possible patients for inclusion are:

- Patients who express their wish to participate in the study on the basis of the information they have received from their treating doctor or the nursing staff at the department of psychiatry.
- Patients who have been identified by medical or nursing staff at the department of psychiatry as being suitable for or interested in participating in the study.

These patients will receive written material and oral information about the project if they are interested. The oral information will elucidate the written information. They will also be informed that the treatment will not take place immediately, but at a time when beds are available in the ward in question. The patient will be included 1 week before the date at which a bed is available (see also trial phases). After inclusion, the patients will be asked not to disclose their type of treatment to the interviewer who assesses severity of depression by means of the Hamilton Depression Scale. This will be mentioned in the randomisation letter the patients receive at inclusion.

4.6.3. Inclusion criteria

- - Age between 18 and 70 years (inclusively).
  - Major depression according to DSM-IV diagnostic criteria (APA 1987).
  - Score of 13 or more on the 17-item Hamilton Depression Scale (Paykel 1990).

4.6.4. Exclusion criteria

- - Clinically dominant psychic or somatic anxiety corresponding to a score of 3 or more on items 10 and 11 (score range from 0 to 4) of the Hamilton Depression Scale (HAM-D_17_).
  - Clinically domonating suicidal ideation corresponding to a score of 3 or more (score range from 0 to 4) on item 3 of the Hamilton Depression Scale (HAM-D_17_), or if patient or investigator are not certain about the degree of suicidality.
  - Alcohol or drug abuse as defined in the M.I.N.I.
  - Epilepsy, as seizures can be triggered by sleep deprivation.
  - Unsafe contraception in women in the reproductive age. ½Safe contraceptive methods are: contraceptive pills, sterilisation, gestagen depot injections, subdermal hormone implantations, hormonal vaginal rings and transdermal depot patches. It is required that fertile women as a minimum use one of these methods over the total study period and two months after the end of the study.
  - Pregnant and lactating women. All women are pregnancy-tested by a urine sample test.
  - Psychotic disorder as defined in the M.I.N.I
  - Antisocial personality disorder as defined in SCID.
  - Borderline personality disorder as defined in SCID.
  - Schizotypal personality disorder as defined in SCID.
  - Clinically significant organic brain disorder or mental retardation causing incapacity.
  - Treatment with antipsychotics
  - Treatment with drugs with a well-known and significant interaction with duloxetine, e.g. isocarboxazide, thioridazine and fluoxetine. Patients in fluoxetine treatment may be included after one week's discontinuation of treatment.
  - Clinically significantly reduced kidney or liver function.
  - ECG abnormalities which may be of clinical significance in connection with the study or study medication.
  - High blood pressure not treated by medication
  - Glaucoma
  - Patients equally fulfilling the M.I.N.I. criteria for major depression and mania or hypomania and thus suffering from a mixed condition.
  - Patients who have a record that makes it doubtful that they can comply with the guidelines of the study (expected low compliance).
  - Patients who due to their medical history or physical tests during the screening procedure were found to have signs of a somatic condition requiring treatment.
  - Patients with bipolar disorder who have not been treated with a mood stabiliser for at least one month.
  - Allergy to filler components of duloxetine tablets.

4.6.5. Criteria for discontinuation of study treatment (end-points)

- The wish to discontinue treatment (sufficient follow-up treatment will be secured). In case of a waiting period treatment will be provided by the Psychiatric Research Unit.
- Patients with intolerable or clinically significant side effects.
- Patients who at any time during the first 9 weeks of the study have score of 15 or more on the Mania Scale (MAS) irrespective of a mania or hypomania diagnosis.
- Patients who display a continued clinical worsening of their depression at two subsequent weekly assessments (first nine weeks of the study).
  - Patients who after the primary hospitalisation experience a worsening of their depression and have to be re-admitted (first 9 weeks).
  - Mania requiring hospitalisation (first 9 weeks).
  - Change of mood stabilizer in bipolar patients (whole study).
  - Patients who do not comply with the single elements of the study will in general be discontinued (but see also "protocol deviations")

4.6.6. Protocol deviations

The following protocol deviations do not mean exclusion from the study:

- - Skipping maximum one of three sleep deprivations in week 2.
  - Not taking up to 15% of the study drug duloxetine (first 9 weeks).
  - Not taking up to 15 % of other prescribed medication. (whole study period).
  - Skipping maximum 2 visits during the first 9 weeks and 2 visits during the following weeks until week 29.

When a patient discontinues participation in the study, this is recorded in the CRF together with the reason for drop-out and the type of follow-up. An individual assessment will decide whether to collect raw data from the last visit (exclusion visit) or from the last previous regular visit.

Collecting last visit data requires that the medicine prescribed has been taken according to the guidelines for compliance.

**4.7. Biochemical and physical tests**

Blood samples are taken at inclusion to be analysed for TSH (thyreoidea stimulating hormone), haemoglobin, creatinine, alanine transferase, alkaline phosphatase, ionised calcium, folic acid, cobalamin and urine hCG (in women). An ECG is made. Group B patients are asked to collect diurnal urine to be examined for cortisol on the first day of hospitalisation and again after 9 weeks, at the end of the study. On the second day of hospitalisation, group B patients give saliva samples, also for cortisol analysis. This provides an opportunity for comparing cortisol in diurnal urine and saliva. The saliva samples are taken in the morning, 15, 30 and 60 minutes after awakening.

Blood pressure is measured at inclusion and after 9 weeks by automatic blood pressure monitor while the study subject is seated. The measurement is repeated several times, until the blood pressure is reproducible. The value from the arm giving the highest result will be used. ECG is made at inclusion and is assessed by a doctor with medical expertise.

**5. COMPLIANCE**

Is ensured by close monitoring of the completion of sleep diary, light diary, exercise diary, and by drug accountability.

Drug accountability:

- Medication prescribed for a non-psychiatric condition (physical illness) is to be administered by the study subjects. Any help in administering the medication is to continue unchanged.
- Drugs prescribed for a psychiatric condition (including hypnotics, anxiolytics and mood stabilisers) have to be brought along at each visit, where the amount taken is checked and recorded in the CRF. The medication given to the patient covers the time until the next visit. For patients who have problems taking their medication, the drug will be distributed into dosing boxes. This medication is thus fully administered by the investigators.

Diaries:

- Compliance to the recommendations regarding sleep, diurnal rhythm, exercise and light are monitored by means of the relevant diaries, which are brought along at each visit. The diaries are kept throughout the whole study.

**6. SOURCE DATA**

The various types of source data are kept as follows:

| Inclusion and exclusion criteria | CRF |
| --- | --- |
| Demographic (anamnestic) data | CRF |
| Blood pressure, height and weight | CRF |
| Concomitant medication | CRF |
| Laboratory tests | Patient record |
| Study medication accounts | CRF |
| Side effects | CRF |
| Primary effect parameters (severity of depression) | CRF |
| Secondary effect parameters (severity of depression) | CRF |
| Tertiary effect parameters (exercise) | CRF |
| Tertiary effect parameters (cortisol) | Patient record |
| Tertiary effect parameters (idiographic method) | CRF |
| Tertiary effect parameters (Preskorn) | CRF |
| Medication (prescribed) | Patient record |

All source data will be kept at Frederiksborg General Hospital, Dept. of Psychiatry, for 15 years.

**7. PATIENT RECORDS**

Patient records are kept with a summary of the patient's case history. For each visit the patient's condition is described, side effects are recorded, and a measurement of severity of depression is made. Skipped visits and the reason for not showing up are also recorded. The patient record is kept separately from a possibly existing patient record at the hospital in a locked room and filed under the patient number.

**8. PATIENT INFORMATION AND INFORMED CONSENT**

Potential study subjects who are interested in information about the study will receive written information about the study and its single elements, e.g. that patients will be hospitalised at the psychiatric hospital for 6 days. An appointment is made with the patient for oral information on the study. Both written and oral information will be given according to the rules on information to and consent by patients, published by the Danish Ministry of Science in 2003, Committee Act on information and consent when involving study subjects in biomedical research projects. (" Act on a Biomedical Research Ethics Committee System and the Processing of Biomedical Research Projects"). See also under selection of patients.

**9. CONTACTS WITH TREATING MEDICAL PROFESSIONALS**

If the patient agrees, short statements will be sent to the referring doctor and the patient's GP, with relevant information about the trial and the outcome for the patient at inclusion and at end of the study. All correspondence with public authorities, among them sick reports and medical certificates, are made by the patient's GP unless the patient explicitly wishes the investigator to do it.

**10. SAFETY ASSESSMENT**

Side effects during treatment are recorded by means of the UKU at each visit. The reporting of side effect is made according to GCP rules requiring that side effects are reported and that a final debriefing report is sent to the Danish Medicines Agency. Thus, all unexpected and severe presumed side effects which are lethal or life-threatening, have to be registered and reported as soon as possible, 7 days after the event has been made known to the investigator at the very latest. Not later than 8 days after the report the investigators will inform the Danish Medicines Agency about follow-up to the report. All other unexpected and serious presumed side effects must be reported 15 days after being made known to the investigator, at the very latest. Each report will be accompanied by comments concerning the possible consequences for the study.

The following definitions will be used according to the guidelines for clinical trials by the Danish Medicines Agency:

"Adverse event: any adverse event experienced by a patient or a study subject in a trial after treatment with a drug, though there may not be any connection between the treatment and the adverse event experienced.

Side effect: any harmful and adverse reaction to a trial drug regardless of dose.

Unexpected side effect: a side effect the severity or character of which are not in agreement with the product information (e.g. the investigator's brochure for a non-licensed trial drug or, if the drug is licensed, the product summary).

Serious adverse event or severe side effect: an event or a severe side effect that irrespective of dosage leads to death, is life-threatening, leads to hospitalisation or extension of current hospitalisation, permanent or serious disability, incapacity for work, or results in congenital anomaly or deformity.

**11. STATISTICS**

To analyse the number of patients with response or remission at three defined points in time (weeks 2, 9 and 29), Chi^2^ distribution is used. To do this, the Last Observation Carried Forward (LOCF) is employed. The assessment of the longitudinal courses of response and remission (and sustained response and remission) uses survival analysis (Kaplan-Meier survival-analysis) including log-rank test to calculate p values (Stassen et al 1993). Furthermore, the Mixed Model Repeated Measures (MMRM) (Brown et al. 1999) will analyse readily available depression scores to determine the difference between the two groups. Thus, MMRM does not use LOCF. The association between cortisol and depression scores and the association between urine and saliva cortisol has been calculated by means of the Spearman correlation coefficient *rho*. The same statistical method will be used to test the association between the various degrees of depression and the effect of sleep deprivation. The idiographic method will be analysed by means of MMRM. Exercise will be analysed as the percentage of compliance to the predefined goal (30 minutes exercise daily). Moreover, the achieved amount of exercise (combined score of the Borg scale and the amount of performed exercise) will be graded into light/moderate/intensive in follow-up phase I. It will be compared with the degree of response and remission in week 29 and analysed by Chi^2^ distribution. The number of study subjects has been calculated on the basis of an expected depression score of 24 on the 17-item Hamilton Depression Scale at inclusion into the study for both groups. After 9 weeks of treatment a decrease to 10 in group A and to 13 in group B is expected. The expected standard deviation is 4. With a type-I error of 0.05 and 0.90 power, 39 patients are required for each group.

No interim analyses have been planned.

GCP guidelines permit that patients may be excluded from the data analysis after the end of the study, if eligibility violations have taken place and if this was not known during the project. This may refer patients who have fulfilled the inclusion criteria, but have not taken any of the study medication, or have taken other prescriptions without telling the investigator, and where these violations were not known until the end of the study. All other patients assessed at the time of inclusion in the study are included in the statistical analyses according to the intention-to-treat method.

**12. DIMENSION**

According to the expected results of the study (see power calculation in the statistics section) and taking into account the possibility to analyse subgroups such as unipolar and bipolar patients, it is intended to include 100 patients into the study, 50 to each group. The patients are included consecutively and it will thus not be possible to foretell how many bipolars and how many unipolars will have been included at the end of the study.

**13. TIME FRAME**

The first patient is expected to enter the study 1 August 2005. This may be postponed, conditional to the duration of procedures in the Danish Medicines Agency, The Data protection Agency and the Scientific Ethics Committee. The last patient is expected to be included 1 July 2007. Data entries are made consecutively and statistical analyses will be made immediately after the last patient has finished the study programme (January 2008). Immediately after this, work on the publication will be commenced.

**14. ACCESS TO SOURCE DATA AND OTHER DOCUMENTS PERTAINING TO THE STUDY**

The investigator grants access to all documents pertaining to the study for study monitors, ethic committee inspectors/audit , the Danish Medicines Agency and the Data Protection Agency.

**15. DATA HANDLING AND MANAGEMENT**

The documents to follow each study subject are:

- Case Report Forms (CRF)
- Patient Record
- Entry form with patient no. and personal ID.
- Patient card

The patient record is completed as required. A study entry form is filled in containing information about referrer, patient address, names and telephone numbers of the closest family, personal ID and patient number. At each visit, all necessary data are entered into the CRF under patient no., initials and date. The patient receives a patient card containing the name of the project (KRONOS), the investigators' names and phone numbers, the name of the study drug (duloxetine) and a brief description of the study. Furthermore, the card has a space for the dates and times of the appointments.

All documents are kept in a locked place, and the entry form is stored separately from the other documents, also in a locked place. All forms are filled in consecutively at patient visits. The monitor has full access to the documents and lacking data are to be retrieved at monitoring visits. The CRF are sent consecutively to the data manger, while a copy stays at the Research Unit. After finishing the study, all documents will be stored in a locked store room on the hospital ground and belonging to the Research Unit, where they will be open to access for 15 years after the end of the study.

**16. GOOD CLINICAL PRACTICE (GCP)**

The trial follows ICH-GCP (EMEA 1997) procedures. The principal investigator has a GCP diploma (Lif 2004).

**17. LABELLING**

The labels of the duloxetine packages contain the following information:

- For a Clinical Trial
- Kronos EudraCT 2005-001855-39
- Batch number (already on the package upon receipt)
- Storing (already on the package upon receipt)
- Responsible doctor: Klaus Martiny, Psychiatric Research Unit, Dyrehavevej 48, 3400 Hilleroed, Denmark
- Phone number 4829-3315
- Capsules Duloxetine 60 mg (against depression) to be taken with water
- Patient number:
- To be stored out of reach for children
- Take your medication with you at each study visit
- Dose and date

The 30 mg Duloxetine capsules will be labelled accordingly.

**18. INVESTIGATOR'S BROCHURE AND PROJECT FILES**

An investigator's brochure will be kept at the investigator's office, containing relevant publications concerning the chronobiological methods used and information about duloxetine. The latter will be delivered by the duloxetine producer.

The project files will contain copies of the study protocol and any protocol amendments, copies of all scales used in the study, and all contracts and agreements with monitor, data manager and statistician and all approval documents of the study. Moreover, the project files will contain monitoring reports.

**19. MONITORING**

Monitoring is done by the GCP Unit of Gentofte Hospital, Niels Andersens Vej 65, 2900 Hellerup, Tel. 3977-7418. Payment is made according to the financial agreement between Frederiksborg County and the GCP Unit.

**20. ETHICAL CONSIDERATIONS**

The following items of concern in regard to ethical consideration are described below.

**20.1. Switch of depression drug**

Most patients to be included in the study are expected to be in pharmacological antidepressive treatment. These patients may therefore have to change from their current medication to a new medication unknown to them. Duloxetine has been licensed in Denmark quite recently and only few patients may have experiences with this drug. The investigator, therefore, has thoroughly explored the side-effect profile of the drug, made on the basis of experiences in countries where it has been used for a longer period of time. The available data show that the drug can be expected to have the same type of side effect as other antidepressants with the same mode of action (serotonin and noradrenaline reuptake inhibitors, SNRI), though with a lesser tendency to sweating and affecting blood pressure. As far as the switch from one medication to the other is concerned, it has clinically been shown that this switch from current medication to duloxetine can be made without slow withdrawal and with a full dose of duloxetine from day one. The inclusion criteria have taken into account those patients who are treated with isocarboxacide and fluoxetine. No worsening of the depression is anticipated owing to the replacement of one medication with the other, though it cannot be totally ruled out that single patients may experience transient mood swings of the kind that are seen from time to time in association with a change of antidepressants. The patients will be informed about this risk, both orally and in the written patient information.

**20.2 . Sleep deprivation**

As previously mentioned, it is a well-known fact that patients responding to sleep deprivation may have a partial or total relapse to depression after the following night's sleep. The study tries to prevent this by means of light treatment, antidepressants and sleep-phase-advance. Bipolar patients are required to be in mood-stabilising treatment to prevent mood swings. The patients will be informed about this risk, both orally and in the written patient information. Over the past year, the Psychiatric Research Unit has used sleep deprivation treatment without any problems of this kind. A literature review shows that the method can be used without causing any serious problems for the patient. It is essential to inform the patients about possible side effects. At a study visit in Milano, at the Villa Turro Clinic run by Professor Francesco Benedetti, the study main investigator and a member of the project group could examine the use of the method. Sleep deprivation is used routinely in this clinic, and it was demonstrated that the method was easy to use, staff was interviewed, and a patient was both interviewed and observed during the night during sleep deprivation.

The Psychiatric Research Unit is member of an international group called Clinical Chronotherapeutics in Affective Disorders, who conduct research in chronobiological methods, sleep deprivation included, and communicate their knowledge about these methods by publications and oral presentations. The Psychiatric Research Unit has gained much of their knowledge through the experiences of this group.

To ensure sufficient support to the patients during the project, the patients may contact the investigator on all week days when hospitalised and on all days of the week when not hospitalised. In addition to this the investigators will be in contact with the patient's contact person in the ward and the treating physician.

For the use by the investigators, an information sheet is prepared containing relevant information about each patient such as phone numbers of the ward, names of contact persons, responsible doctor, GP, and closest relative. This sheet is filed in the patient record. The patient receives a patient card with e.g. the investigators' cell phone numbers which the patients can call at any time during working hours and other relevant information.

Outside working hours, during hospitalisation, the patients are asked to contact the staff in the ward. Outside hospitalisations the patient should contact the doctor on call or the psychiatric emergency room who will be informed about the project.

During sleep deprivation in weeks 4, 5 or 6 the patients will be hospitalised in an open ward. However, they will be allowed to leave the ward during daytime and thus only have to stay in the ward for sleep deprivation. These sleep deprivations are thus not different from the one in week 2, after which the patients are discharged in the following morning. In weeks 4 and 5 the patients will be familiar with the procedure and the investigators will know how the patients react to sleep deprivation. It has therefore been considered justifiable to let the patients have a break after sleep deprivation. Before discharge, the patients will be assessed in the ward.

In general, patients who unexpectedly get worse after sleep deprivation and who should have been discharged can stay in hospital as long as necessary.

**20.3 Time of inclusion**

The bed situation in a ward can change suddenly due to very ill patients needing immediate hospitalisation. This means that hospitalisation of a potential study subject who has agreed to participation in the study may be postponed for a short period until there is a vacant bed. As patients with depression often have been ill for many months, we do not regard a postponement of up to 2 weeks as a problem, in particular because the Psychiatric Research Unit during the waiting period will make certain the patient receives the proper treatment, either in the Unit or in a relevant specialist practice. We think it unlikely that a waiting period of more than 2 weeks could occur. The regional head of psychiatry has approved that study subjects primarily be admitted to their local psychiatric wards. Hospitalisation in other open wards but the local ones may only occur in connection with atypical situations and after discussion with the regional head of psychiatry. Thus, the study can make use of 2 open wards per study subject. If the waiting period exceeds 2 weeks, an assessment will be made taking into consideration whether the potential study subject still wishes to enter the study and whether a longer waiting period can be justified from a therapeutic point-of-view. If a potential study subject is very ill due to depression or the depression has worsened during the waiting period, the patient will be advised not to enter the study in order not to delay an envisaged change of the patient's treatment. The problem is mentioned in the written patient information leaflet.

**21. FINANCING AND INSURANCE**

The study is partly supported by an "unrestricted grant" by Eli Lilly Denmark, with a maximum amount of DKK 1,500,000. The contract made with Eli Lilly places full responsibility for the project on the Psychiatric Research Unit in Hilleroed. The Psychiatric Research Unit remains completely independent of Eli Lilly Denmark. Applications to cover the remaining part of the budget will continuously be made to research foundations.

The patients will be covered by a patient insurance and the product liability insurance by Eli Lilly Denmark.

Duloxetine will be delivered and paid throughout the complete study period of 29 weeks. If a patient is switched to another antidepressant during the study, this drug is paid by the project during the study period.

It should finally be mentioned that the project has allocated funds to 2 full investigator employments over the period of 2 years.

**22. GUIDELINES FOR PUBLICATION**

First and last authors will be Klaus Martiny and Per Bech. All others involved in the project will be co-authors, corresponding to the amount of their involvement as to concept, workload or analyses. The authors will aim at publishing the results in peer-reviewed international journals and present them at Danish and international meetings and congresses.

**23. REPORT**

After finishing the study, the sponsor will inform the Danish Medicines Agency within 90 days of the termination of the study and submit the study results to the Agency as soon as possible hereafter, including the number of treated patients, dosages used, duration of dosages, achieved results and observed side effects. Annual lists of presumed serious adverse events occurred during the study period will be compiled and sent to the Danish Medicines Agency together with a report on the study participants' safety.

**24. NOTIFICATION TO THE STUDY DRUG MANUFACTURER**

When the Investigator/Sponsor submits the study protocol to the Danish Medicines Agency, Eli Lilly Denmark will be informed of the application at the same time.

**25. REFERENCES**

Akiskal HS, Brieger P, Mundt C, Angst J, Marneros A. Temperament und affektive Störungen. Nervenarzt 2002; 73: 262-271*.*

American Psychiatric Association. Diagnostic and Statistical Manual of Mental Disorders. 4th edition (DSM-IV). American Psychiatric Association, Washington DC,1994.

Angst J, Gamma A, Sellaro R, Lavori PW, Zhang H. Recurrence of bipolar disorders and major depression. A life-long perspective. Eur Arch Psychiatry Clin Neurosci 200; 253:236-240.

Babyak M, Blumenthal JA, Herman S, Khatri P, Doraiswamy M, Moore K, Craighead WE, Baldewicz TT, Krishnan KR. Exercise treatment for major depression: maintenance of therapeutic benefit at 10 months. Psychosom Med. 2000 Sep-Oct;62(5):633-8.

Bech P, Bille J, Waarst S, Wiese M, Borberg L, Treufeldt P, Kessing L. Validity of HONOS profiles in identifying frequently hospitalised patients with mental disorders 2005 (submitted).

Bech P, KastrupM, Rafaelsen OJ. Mini-compendium of rating scales for states of anxiety, depression, mania, and schizophrenia with corresponding DSM-III syndromes. Acta Psychiatr Scand 1986: 73, Suppl 326: 7-37.

Bech P, Rasmussen NA, Olsen LR, Noerholm V, Abildgaard W. The sensitivity and specificity of the Major Depression Inventory, using the Present State Examination as the index of diagnostic validity. J Affect Disord 2001; 66: 159-164.

Bech P, Shapiro RW, Sihm F, Nielsen BM, Sorensen B, Rafaelsen OJ. Personality in unipolar and bipolar manic-melancholic patients. Acta Psychiatr Scand 1980; 62: 245-257.

Bech P. The Bech-Rafaelsen Mania Scale (MAS) in clinical trials for bipolar disorder. A twenty-year review of its use as an outcome measure. CNS Drugs 2002(b); 16: 47-63

Bech P. The Bech-Rafaelsen Melancholia Scale (MES) in clinical trials of therapies in depressive disorders: a 20-year review of its use as outcome measure. Acta Psychiatr Scand 2002(a): 106: 252-264.

Bech P. Measuring the dimensions of psychological general well-being by the WHO-5. QoL Newsletter 2004; 32: 15-16.

Benedetti F, Barbini B, Campori E, Fulgosi MC, Pontiggia A, Colombo C. Sleep phase advance and lithium to sustain the antidepressant effect of total sleep deprivation in bipolar depression: new findings supporting the internal coincidence model? J Psychiatr Res. 2001; 35:323-9.

Benedetti F, Colombo C, Pontiggia A, Bernasconi A, Florita, M, Smeraldi E. Morning light treatment hastens the antidepressant effect of citalopram: a placebo-controlled trial. J Clin Psychiatry 2003; 64, 648-653.

Berger M, van Calker D, Riemann D. Sleep and manipulations of the sleep-wake rhythm in depression. Acta Psychiatr Scand 2003;(Suppl 418):83-91.

Blumenthal JA, Babyak MA, Moore KA, Craighead WA, Herman S, Khatri P, Waugh R, Napolitano MA, Doraiswami PM, Krishnan KR. Effects of exercise training on older adults with major depression. Arch Intern Med 1999; 159: 2349–56.Brown, H., Prescott, R. Applied mixed models in medicine New York: Wiley 1999.

Buxton OM, Lee CW, L'Hermite-Baleriaux M, Turek FW, Van Cauter E. Exercise elicits phase shifts and acute alterations of melatonin that vary with circadian phase. Am J Physiol Regul Integr Comp Physiol. 2003 Mar; 284(3):R714-24.

Colombo C, Benedetti F, Barbini B, Campori E, Smeraldi E. Rate of switch from depression into mania after therapeutic sleep deprivation in bipolar depression. Psychiatry Res. 1999;86:267-270.

Colombo C, Lucca A, Benedetti F, Barbini B, Campori E, Smeraldi E. Total sleep deprivation combined with lithium and light therapy in the treatment of bipolar depression: replication of main effects and interaction. Psychiatry Res 2000;95:43-53.

Detke MJ, Lu Y, Goldstein DJ, McNamara RK, Demitrack MA. Duloxetine 60 mg once daily dosing versus placebo in the acute treatment of major depression. J Psychiatry Res 2002; 36: 383-390.

EMEA. European Medicines Agency. Note for guidance on good clinical practice. London: EMEA 1997 (CPMP/ICH/135/95)

First MB. SCID-II. Odense: Syddansk Universitetsforlag 2002.

Fritzsche M, Heller R, Hill H, Kick H. Sleep deprivation as a predictor of response to light therapy in major depression. J Aff Disord 2001; 62: 207-215.

Goodwin GM, Evidence-based guidelines for treating bipolar disorder: recommendations from the British Association for Psychopharmacology. Consensus Group of the British Association for Psychopharmacology. J Psychopharmacol. 2003;17:149-173.

Goodwin GM. Evidence-based guidelines for treating bipolar disorders: recommendations from the British Association for Psychopharmacology. J Psychopharmacology 2003; 17: 149-173.

Horne JA, Ostberg O. A self-assessment questionnaire to determine morningness-eveningness in human circadian rhythms. Int J Chronobiol. 1976;4:97-110.

Horne JA, Ostberg O. Individual differences in human circadian rhythms. Biol Psychol. 1977;5:179-190.

Kasper S, Wehr TA, Bartko JJ, Gaist PA, Rosenthal NE 1989. Epidemiological findings of seasonal changes in mood and behaviour: a telephone survey of Montgomery County, Maryland. Archives of General Psychiatry 46:823-33.

Keller MB. Improving the course of illness and promoting continuation of treatment of bipolar disorder. J Clin Psychiatry. 2004;65 (Suppl 15):10-4.

Kelly G.A. The psychology of personal conctructs. 1955; NewYork: Norton.

Kennedy N, Abbott R, Paykel ES. Remission and recurrence of depression in the maintenance era: long-term outcome in a Cambridge cohort. Psychol Med. 2003 Jul;33(5):827-38. Department of Psychiatry, University of Cambridge, Addenbrooke's Hospital.

Kessing LV, Hansen MG, Andersen PK. Course of illness in depressive and bipolar disorders. Naturalistic study, 1994-1999. Br J Psychiatry. 2004;185:372-377.

Kuhs H, Tölle R. Sleep deprivation therapy. Biol Psychiatry 1991; 29: 1129-1148.

Kvist J, Kirkegaard C. Effect of repeated sleep deprivation on clinical symptoms and the TRH test in endogenous depression. Acta Psychiatr Scand. 1980 Nov;62(5):494-502.

Larsen JK, Lindberg ML, Skovgaard B. Sleep deprivation as treatment for endogenous depression.. Acta Psychiatr Scand. 1976 Sep;54(3):167-73.

Licht RW, Qvitzau S, Allerup P, Bech P. Validation of the Bech-Rafaelsen Melancholia Scale and the Hamilton Depression Scale in patients with major depression; is the total score a valid measure of illness severity? Acta Psychiatr Scand. 2005 Feb;111(2):144-9.

Lingjærde O, Ahlfors UG, Bech P, Dencker SJ, Elgen K. The UKU side effect rating scale. A new comprehensive rating scale for psychotropic drugs and a cross-sectional study of side effects in neuroleptic-treated patients. Acta Psychiatr. Scand 1987: 76 (suppl.334).

Lov om videnskabsetisk komitésystem og behandling af biomedicinske forskningsprojekter. Videnskabsministeriet, 2003.

Loving RT, Kripke DF, Shuchter SR. Bright light augments antidepressant effects of medication and wake therapy. Depress Anxiety. 2002;16:1-3.

Martiny K. Adjunctive bright light in non-seasonal major depression. Acta Psychiatr Scand 2004;110 (Suppl 425):7-28.

Neumeister A, Goessler R, Lucht M, Kapitany T, Bamas C, Kasper S. Bright light therapy stabilizes the antidepressant effect of partial sleep deprivation. Biological Psychiatry 1996; 3: 16-21.

O’Sullivan RL, Fava M, Agustin C, Baer L, Rosenbaum JF. Sensitivity of the six item Hamilton Depression Rating Scale. Acta Psychiatr Scand 1997: 95: 379-384.

Olsen LR, Jensen DV, Noerholm V, Martiny K, Bech P. The internal and external validity of the Major Depression Inventory in measuring severity of depressive states. Psychol Med 2003; 33: 351-356.Olsen LR, Mortensen EL, Bech P. The SCL-90 and SCL-90R versions validated by item response models in a Danish community sample. Acta Psychiatr Scand 2004; 110: 225-229.

Ostenfeld I. Abstinence from night sleep as a treatment for endogenous depressions. The earliest observations in a Danish mental hospital (1954) and an analysis of the causal mechanism. Dan Med Bull. 1986;33:45-49.

Ostenfeld I. Treatment of endogenous depression by deprivation of sleep. Ugeskr Laeger. 1973 6;135:2632-2633.

Paykel ES. Use of the Hamilton Depression Rating Scale in general practice. In: Bech P, Coppen A (eds.) The Hamilton Scales. Berlin: Springer 1990, pp 159-178

Raskin J, Goldstein DJ, Mallinckrodt CH, Ferguson MB. Duloxetine in the long-term treatment of major depressive disorder. J Clin Psychiatry 2003; 64: 1237-1244.

Schilgen B, Tölle R. Partial sleep deprivation as therapy for depression. Arch Gen Psychiatry 1987; 37: 267-271.

Sheehan DV, Lecrubier Y, Harnett Sheehan K, Amorim P, Janavs J, Weiller E, Hergueta T, Baker R, Dunbar G. The Mini International Neuropsychiatric Interview (M.I.N.I.): The development and validation of a structured diagnostic psychiatric interview. J Clin Psychiatry 1998; 59 (Suppl. 20): 22-33.

Sokero TP, Melartin TK, Rytsala HJ, Leskela US, Lestela-Mielonen PS, Isometsa ET. Prospective study of risk factors for attempted suicide among patients with DSM-IV major depressive disorder. Br J Psychiatry. 2005 Apr;186:314-8.

Smeraldi E, Benedetti F, Barbini B, Campori E, Colombo C. Sustained antidepressant effect of sleep deprivation combined with pindolol in bipolar depression. A placebo-controlled trial. Neuro-psychopharmacology 1999;20:380-5.

Stassen HH, Delini-Stula A, Angst J. Time course of improvement under antidepressant treatment. A survival analytical approach. Eur Neuropsychopharmacol 1993; 3: 127-135.

Sundhedsstyrelsens kampagne ”Rør dig 30 minutter om dagen”, 2004 .Evaluerings rapport på: http://www.sst.dk/publ/Publ2004/Eval30minkamp2004.pdf

Svendsen K. Sleep deprivation therapy in depression. Acta Psychiatr Scand. 1976 Sep;54(3):184-92.

Szuba MP, Baxter LR Jr, Altshuler LL, Allen EM, Guze BH, Schwartz JM, Liston EH. Lithium sustains the acute antidepressant effects of sleep deprivation: preliminary findings from a controlled study. Psychiatry Res. 1994;51:283-295.

Terman M, White TM, Jacobs J. Automated Morningness-Eveningness Questionaire. Self-assessment version. New York, Center for Environmental Therapeutics, 2002. Accessed 11 April 2005 at [http://www.cet.org](http://www.cet.org/).

Terman M, Macchi MM, Goel N, Rifkin JB, Terman JS, Williams JBW. Diagnostic Reliability and Symptom Pattern of DSM-IV Atypical Features Seasonal and Nonseasonal Depression. Chronobiology International 2003;20:1157-1159.

Thunedborg K. Beyond the Hamilton depression scores in long-term treatment of manic-melancholic patients: prediction of recurrence of depression by quality of life measurements. Psychother Psychosom. 1995; 64: 131-140.

Voderholzer U, Valerius G, Schaerer L, Riemann D, Giedke H, Schwarzler F, Berger M, Wiegand M. Is the antidepressive effect of sleep deprivation stabilized by a three day phase advance of the sleep period? A pilot study. Eur Arch Psychiatry Clin Neurosci. 2003;253:68-72.

Vovin RI, Fakturovich AI. [Sleep deprivation as a method of treating endogenous depression] Zh Nevropatol Psikhiatr Im S S Korsakova. 1985;85:560-565.

Wirz-Justice A, Terman M, Oren DA, Goodwin FC, Kripke DF, Whybrow PC, Wisner KL, Wu JC, Lam RW, Berger M, Danilenko KV, Kasper S, Smeraldi E, Takahashi K, Thompson C, Hoofdakker R. Brightening depression (letter to the editor). Science 2004; 303:467-468.

Wirz-Justice A, Van den Hoofdakker RH. Sleep deprivation in depression: what do we know, where do we go? Biol Psychiatry. 1999;46:445-453.

Wirz-Justice A. Personal communication regarding unpublished investigations on the influence of exercise on the sleep-wake cycle 2005.
