# Supplementary material for: The Day-to-Day Acute Effect of Wake Therapy in Patients with Major Depression Using the HAM-D6 as Primary Outcome Measure: Results from a Randomised Controlled Trial
Source: PLoS One. 2013 Jun 28;8(6):e67264. doi: 10.1371/journal.pone.0067264 (PMC3696105; doi:10.1371/journal.pone.0067264)
Supplement: Supplementary file 7 [file pone.0067264.s002.doc]

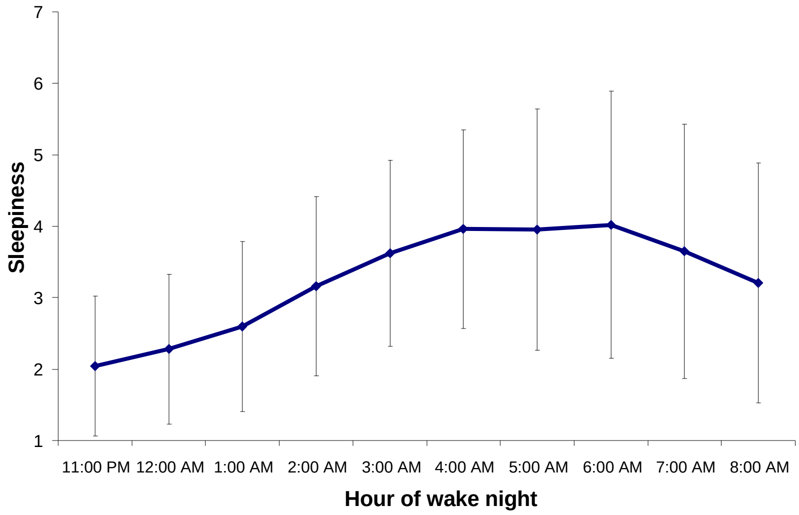

Acute effect of wake therapy
